# Venomics of the ectoparasitoid wasp *Bracon nigricans*

**DOI:** 10.1186/s12864-019-6396-4

**Published:** 2020-01-10

**Authors:** Andrea Becchimanzi, Maddalena Avolio, Hamed Bostan, Chiara Colantuono, Flora Cozzolino, Donato Mancini, Maria Luisa Chiusano, Pietro Pucci, Silvia Caccia, Francesco Pennacchio

**Affiliations:** 1 Department of Agricultural Sciences, University of Napoli Federico II, Portici, NA Italy; 2 Department of Chemical Sciences and CEINGE Biotecnologie Avanzate, University of Napoli Federico II, Napoli, Italy; 3 Present address: Plants for Human Health Institute, North Carolina State University, Kannapolis, NC USA; 4 Present address: Infrastrutture di Ricerca per le Risorse Biologiche Marine, Stazione Zoologica Anton Dohrn, Villa Comunale, 80121 Napoli, Italy

**Keywords:** Idiobiont parasitoids, Host-parasitoid interactions, Venom proteome, Venom transcriptome, Host regulation

## Abstract

**Background:**

Venom is one of the most important sources of regulation factors used by parasitic Hymenoptera to redirect host physiology in favour of the developing offspring. This has stimulated a number of studies, both at functional and “omics” level, which, however, are still quite limited for ectophagous parasitoids that permanently paralyze and suppress their victims (i.e., idiobiont parasitoids).

**Results:**

Here we present a combined transcriptomic and proteomic study of the venom of the generalist idiobiont wasp *Bracon nigricans*, an ectophagous larval parasitoid of different lepidopteran species, for which we recently described the host regulation strategy and the functional role of the venom in the induction of physiological changes in parasitized hosts. The experimental approach used led to the identification of the main components of *B. nigricans* venom involved in host regulation. Enzymes degrading lipids, proteins and carbohydrates are likely involved in the mobilization of storage nutrients from the fat body and may concurrently be responsible for the release of neurotoxic fatty acids inducing paralysis, and for the modulation of host immune responses.

**Conclusion:**

The present work contributes to fill the gap of knowledge on venom composition in ectoparasitoid wasps, and, along with our previous physiological study on this species, provides the foundation on which to develop a functional model of host regulation, based both on physiological and molecular data. This paves the way towards a better understanding of parasitism evolution in the basal lineages of Hymenoptera and to the possible exploitation of venom as source of bioinsecticidal molecules.

## Background

Parasitic Hymenoptera are part of one of the most speciose insect orders, which includes the largest number of insect natural enemies [1]. This group of insects exhibit an impressive diversity of adaptive strategies to regulate host physiology [2]. More basal lineages include ectoparasitoids, whose larvae feed externally on the host’s body [3]. In most cases, these wasps have a fairly broad host range and are idiobionts, since they induce a rapid paralysis of the host which is quickly exploited as a static source of nutrients [4, 5]. In contrast, koinobionts, which in most cases develop as endophagous parasitoids (i.e., inside the body of the host), show more complex and subtle host regulation strategies, allowing a prolonged interaction of its juveniles with a living host [2, 6]. However, in both cases the growth and development of parasitoids is dependent on the physiological regulation of the host, mediated by a wide range of parasitic factors, some of which are present both in ectoparasitic and endoparasitic wasps (i.e., venom and larval secretions), while others only occur in endoparasitoids (e.g., polydnaviruses and teratocytes) [2, 7–10].

The most common and widely studied source of host regulation factors is the venom: a complex blend of proteinaceous and non-proteinaceous compounds injected at the oviposition in the host by the parasitoid female [2]. The role and composition of venom reflect the different lifestyles of ecto- and endoparasitic wasps. In general, the venom from ectoparasitoids is involved in the rapid host immobilization, to facilitate food uptake by their larvae, while venom from endoparasitoids triggers a very diverse set of alterations, interfering with the host immune system and development or synergizing the effects of other maternal factors introduced into the host (e.g., polydnaviruses) [11].

The large number of studies on venom of endoparasitoid wasps has led to the identification and functional characterization of several molecules involved in the host regulation [12–19]. More recently, the advent of high-throughput technologies greatly contributed to this research area through a “multi-omic” approach often denoted as venomics, which is the integration of genomics, transcriptomics and proteomics [20–27]. Such an integrated approach provides a remarkable amount of molecular information and paves the way for the identification and exploitation of new biomolecules potentially useful for therapeutic and agricultural applications [28].

In contrast, only few venom components have been identified from a limited number of ectoparasitoids, despite the abundance of known species [11]. A significant part of the research efforts are focused on host paralysis, which is the most evident and dramatic symptom induced by the venom of ectoparasitic wasps. The venom from *Ampulex compressa* contains components with both pre- and post-synaptic effects on GABA-gated chloride channels, determining host paralysis [29]. The venom of *Philanthus triangulum* contains neurotoxic compounds (philantotoxins) which affect both the central and the peripheral nervous system, blocking the neuromuscular transmission [30]. Envenomation by *Liris niger* causes host paralysis, due to blockage of synaptic transmission, but the venom components are still uncharacterized [31]. The venoms of *Bracon* (=*Habrobracon*; =*Microbracon*) *hebetor* and *Eupelmus orientalis* cause a permanent paralysis, likely triggered by the neurotoxic activity of phospholipases [32–34].

Studies on other host regulation properties of ectoparasitoid venom are limited, with the exception of the pupal ectoparasitoid *Nasonia vitripennis*, for which a more comprehensive analysis of host alterations has been carried out, taking into consideration developmental arrest, metabolic changes and immunosuppression [35–37]. The main components of this venom [38] include, among others, serine- and metalloproteases, triggering apoptotic processes in *Spodoptera frugiperda* Sf21 cell line [39], two serpins and another protease inhibitor, interfering with prophenoloxidase activation in the host *Musca domestica* [40, 41], and a chitinase, inducing an upregulation of host genes involved in the immune response against fungi [42]. Additional functional studies will be likely fostered in this research area since *N. vitripennis* is a powerful model system, for which the genome sequence and molecular tools are available [43, 44].

High-throughput technologies contributed to explore, to a limited extent, the venom composition of other ectoparasitic wasps, such as *Anisopteromalus calandrae* [45], *Scleroderma guani* [46] and *B. hebetor* [47]. This latter generalist species that parasitizes a number of moth larvae was one of the first studied for its venom composition, which includes neurotoxic proteinaceus components, only partially characterized [33, 48–51].

Here we present a venomic study on a congeneric species*,* the generalist idiobiont wasp *Bracon nigricans*, which, like *B. hebetor*, is an ectophagous larval parasitoid of several lepidopteran species [52, 53]. This offers the opportunity to compare the molecular toolkit used by two related generalist wasp species, attacking lepidopteran hosts. The integration of transcriptomics and proteomics aims at identifying the venom components of *B. nigricans* responsible for the observed effects on the laboratory host *Spodoptera littoralis*, which include a reduced immune competence and fat body degradation associated with an enhanced host nutritional suitability for the feeding wasp larvae [54]. The present study sheds light on the identity of venom molecules involved in the host regulation strategy adopted by *B. nigricans*, providing (i) the information required to develop the molecular details of the physiological model of host-parasitoid interaction recently proposed [54], (ii) evolutionary insights on venom blend diversification in related species with a similar host range, (iii) the opportunity to exploit venom components as potential bioinsecticides.

## Results

### Assembly and analysis of venom glands transcriptome

**Fig. 1:**
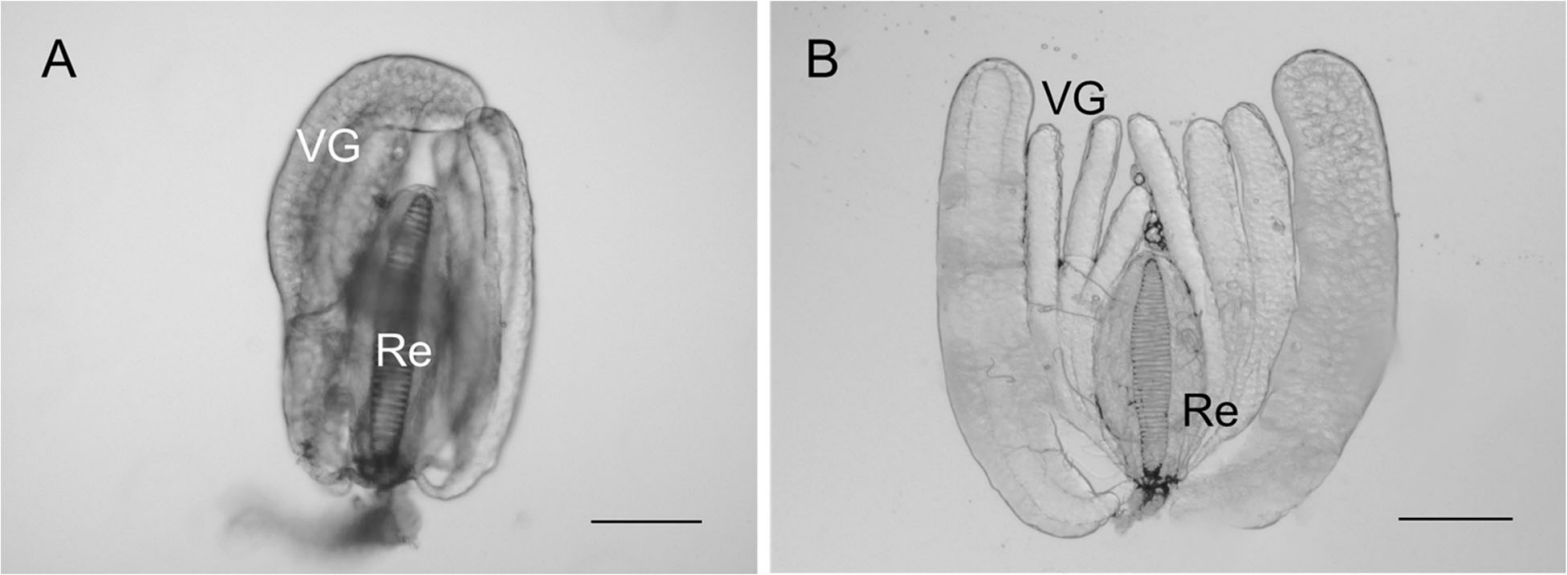
Venom glands and reservoir of *Bracon nigricans*. Venom glands (VG) of *B. nigrican*s tightly stick together around a muscular sac, the venom reservoir (Re) (**a**). When venom glands are separated (**b**), it is evident that they are in total 8, two of which are clearly larger. The helicoidal chitin layer that coats the reservoir cavity is clearly visible (**a**, **b**). Scale bars: 200 μm

In order to obtain the transcriptome profile of *B. nigricans* venom glands (Fig. 1), a cDNA library was constructed and sequenced adopting the Illumina paired-end reads sequencing. The sequencing output consisted of 25,252,591 read pairs that were reduced to 24,437,756 pairs and 796,318 single reads after adapter removal, trimming and quality check. De novo assembly of processed reads by Trinity software resulted in a total of 42,334 transcripts, with their length ranging from 201 to 29,885 bp, and a mean assembled length of 1206.93 bp (N_50_ = 2636 bp). The Trinity assembly output specifically consisted of 25,782 unigenes, each one representing a set of transcripts from the same locus. Main results and features of the assembly are presented in Table 1.

**Table 1:** Overview of the de novo transcriptome assembly of *Bracon nigricans* venom glands

Sequencing and assembly parameters	Value
Total number of raw reads	25,252,591
Paired-end reads after cleaning	24,437,756
Single reads after cleaning	796,318
Collapsed transcripts/unigenes^1^	42,334/25782
N_50_ of unigenes (bp)^2^	2636
% Reads mapped	93.3
Transcripts vs *Bracon nigricans* venom (proteins)^3^	4783 (109)
Transcripts vs UniprotKb (proteins)^3^	22,218 (9915)

^1^The number of transcripts/unigenes resulting after the assembly by Trinity and the subsequent collapsing step by CD-HIT

^2^N_50_ value, which represent the threshold delimiting 50% of the contigs in the entire assembly which are equal to or larger than the reported value

^3^e-value = 1e-5, in brackets the number of unique proteins found

BLASTx similarity search results revealed that about 52.5% of the total assembled transcripts (22,218 sequences) have at least one match with the UniProtKB database. Among these sequences, 520 transcripts show similarities with venom and toxin related proteins deposited in the Tox Prot database. In particular, 455 and 65 transcripts resulted similar to putative venom proteins and putative toxin proteins, respectively.

**Fig. 2:**
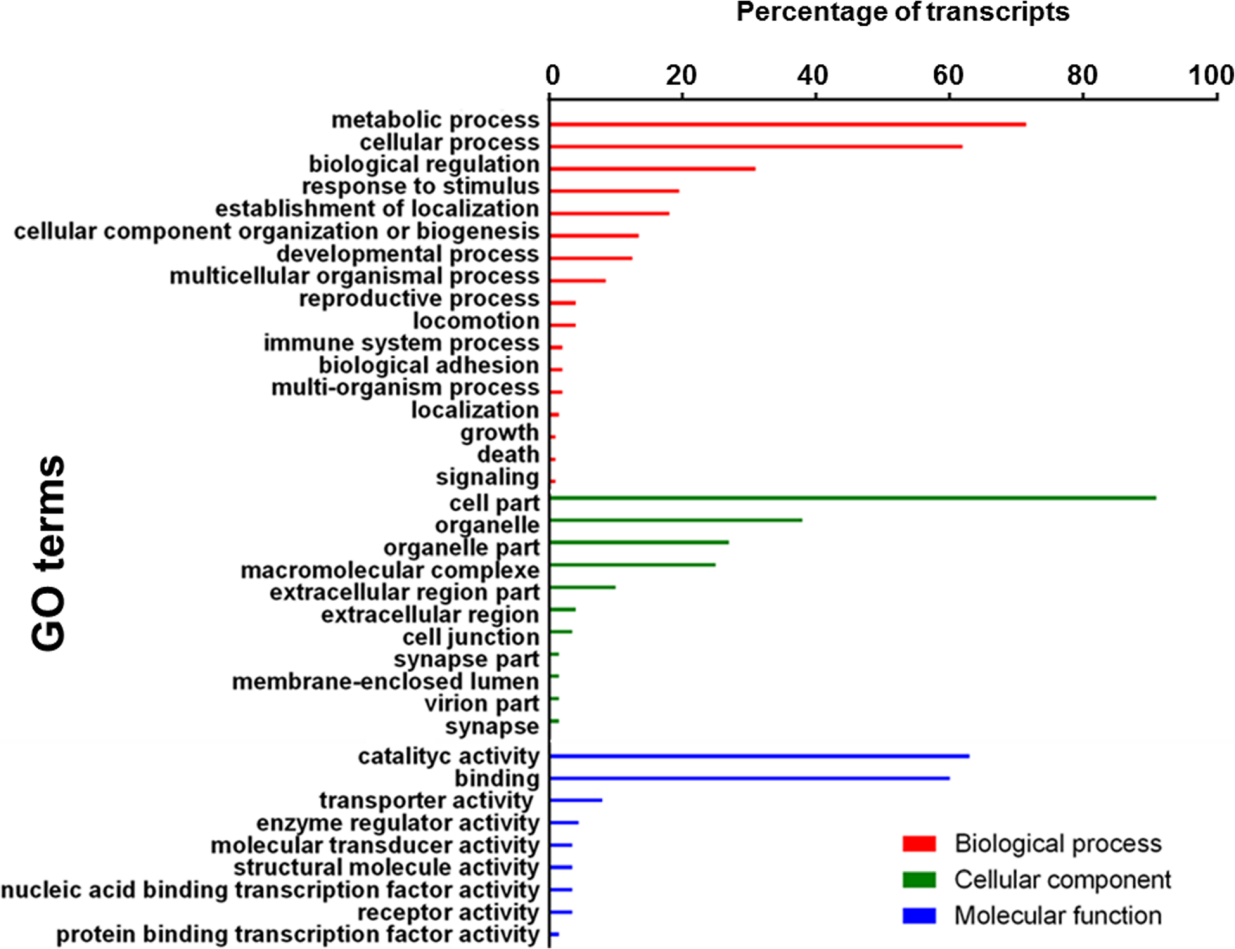
Histograms reporting Gene Ontology (GO) level 2 annotation of the transcripts from *Bracon nigricans* venom glands. GO classification was assigned in relation to three sub-ontologies (biological process, cellular component, and molecular function). For each category, the bars represent the transcripts assigned to a GO term as percentages of the total number of transcripts with GO assignments

The remaining 20,116 (47.5%) transcripts did not match with any other sequence in the UniProtKB database, and 2% of them (387 transcripts) showed a putative signal peptide. The functional annotation of the transcripts, performed using BLAST2GO, shows that 22,222 sequences (52.48% of the total assembly) share significant similarity to proteins with assigned Gene Ontology (GO) terms. These sequences are classified, at level two, into 37 functional subcategories belonging to the three main ontological categories: biological process, cellular component and molecular function. “Metabolic process” and “cellular process” are the dominant GO terms in the biological process category (Fig. 2). Highly represented terms within cellular component are “cell part” and “organelle”, whereas in the molecular function category “catalytic activity” and “binding” are the most represented terms (Fig. 2).

The SignalP analysis led to the identification of 3089 transcripts encoding proteins with a possible signal peptide at the N-terminus, suggesting that the encoded products are destined to the secretory pathway. The overall distribution of transcript abundance, expressed in RPKM, is shown in Fig. S1 (Additional file 1) and the 49 most abundant (RPKM> 100) transcripts corresponding to this subset, are listed in Additional file 2: Table S1. However, this information (i.e., the presence of signal peptide and the transcript abundance) has been subsequently integrated with the proteomic analysis, as described below.

### Identification of venom proteins by proteomic approach

**Fig. 3:**
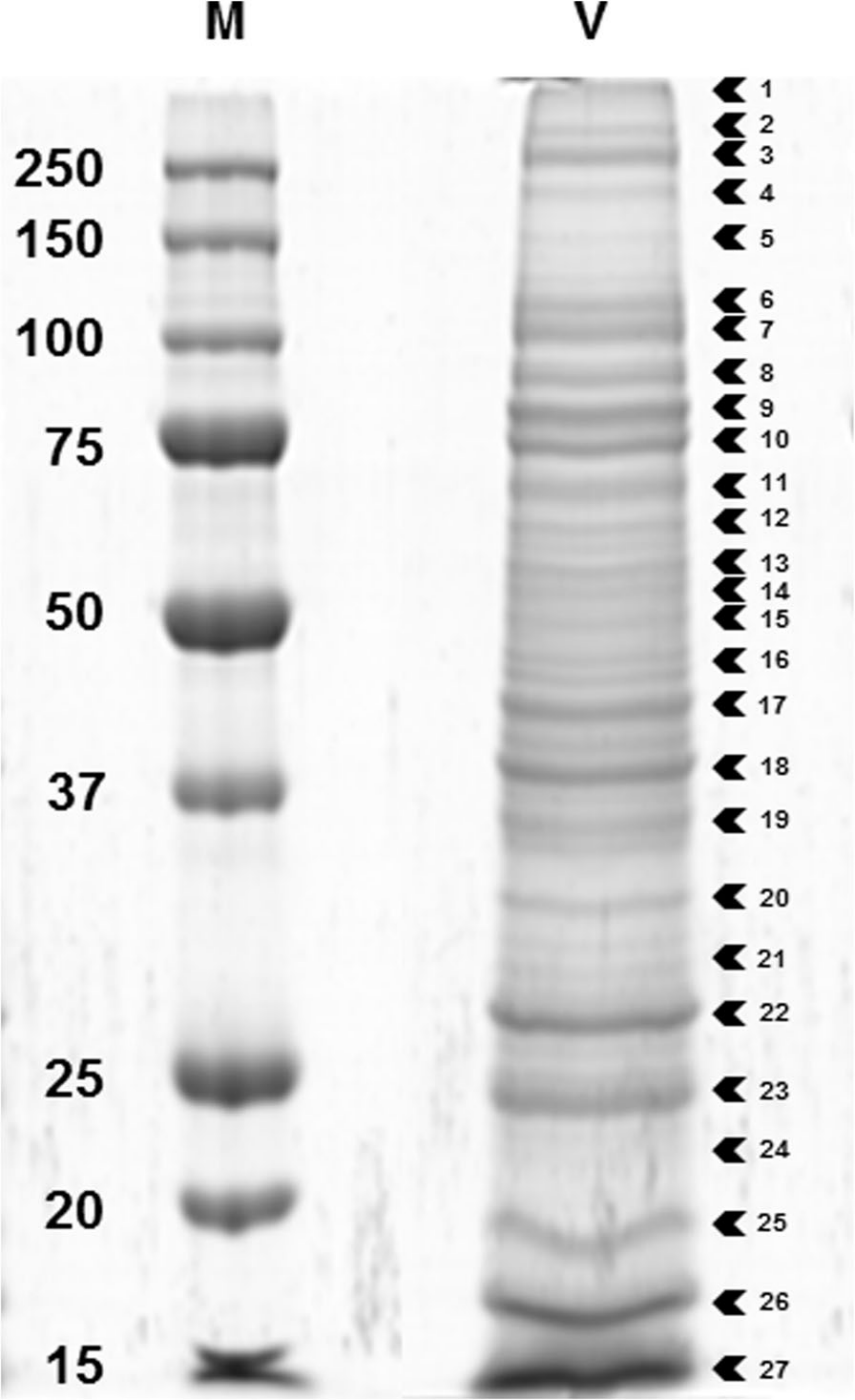
SDS-PAGE of crude venom extract from *Bracon nigricans*. Venom Proteins were separated on a 10% SDS-PAGE gel and stained with Coomassie Brilliant Blue G250 (V). Molecular mass marker is shown on the left with the molecular mass in kDa corresponding to each band (M). Selected protein bands (from 1 to 27) were excised from the gel and processed for LC/MS-MS analysis

Upon separation of *B. nigricans* venom proteins by SDS-PAGE, bands with apparent molecular masses ranging from 15 to 300 kDa were observed. Figure 3 shows the 27 selected protein bands and the relative range of molecular weight (apparent molecular mass). The analyzed bands were resolved into 109 proteins by LC/MS-MS (Additional file 3: Table S2), identified by matching the resulting peptides to the full-length proteins predicted from the venom gland transcriptome assembly. Matches of at least two peptides were considered valid.

### Integration of transcriptomic and proteomic analyses

To identify a robust set of abundant venom proteins, all data were analyzed under the assumption that venom proteins are secreted and highly expressed in the venom glands. This was performed by filtering the proteome for the presence of the signal peptide identified by SignalP tool (approximately 22% of the 109 total proteins) and, among these, we focused on those in the upper third of RPKM values distribution, exceeding the threshold value of 70. This allowed to generate a list of 18 venom proteins highly abundant and, as such, with important functions (Table 2 and Additional file 4: Table S3). Only 10 of these candidates showed similarity with protein sequences deposited in the UniProt/SwissProt database.

**Table 2:** Most representative *Bracon nigricans* venom proteins

Transcript ID	RPKM	Band	Best hit in SwissProt db	InterProScan Result	Species having putative homologs
comp24796_c0_seq1*	11,689.63	27	No hits found	PBP/GOBP family - Odorant Binding Protein	Ac, Ci, Nv, Lh, Pp
comp22364_c1_seq2*	9209.46	26	Phospholipase A2 (PA3A/PA3B/PA5) P16354 *Heloderma suspectum*	Phospholipase A2	Bh, Eo, Pc, Tn
comp17530_c0_seq1	4073.65	27	No hits found	Prokaryotic membrane lipoprotein lipid attachment site profile	Bh
comp24797_c0_seq1*	3996.5	11, 12	Venom carboxylesterase-6 B2D0J5 *Apis mellifera*	Carboxylesterase, type B	Ac, Dc, Hd, Nv
comp24806_c0_seq1	3088.82	23	Venom allergen 5 P86870 *Vespa magnifica*	Venom allergen 5-like - CRISP	Ci, Hd, Lh, Mh, Nv, Tb
comp24842_c0_seq1	436.95	10, 11	No hits found	DUF4803	Cd, Mh, Pc, Pl
comp14282_c0_seq1	398.38	7	No hits found	DUF4803	Cd, Mh, Pc, Pl
comp16818_c0_seq2*	288.36	20	Chymotrypsin-1 Q27289 *Anopheles gambiae*	Serine protease, peptidase, chymotrypsin	Ae, Bh, Ci, Cr, Es, Hd, Nv, Ph, Pp, Tn
comp20898_c0_seq1	280.35	10	No hits found	DUF4803	Cd, Mh, Pc, Pl
comp18097_c0_seq1	224.37	27	No hits found	PBP/GOBP family - Odorant Binding Protein	Ac, Ci, Nv, Lh, Pp
comp20936_c0_seq1	180.95	9	No hits found	DUF4803	Cd, Mh, Pc, Pl
comp13820_c0_seq1*	156.34	6, 7	Aminopeptidase M1-A Q6Z6L4 *Oryza sativa*	Aminopeptidase N-type	Cc, Lb, Ph, Pl
comp24916_c0_seq1	117.70	9, 10	No hits found	DUF4803	Cd, Mh, Pc, Pl
comp21345_c1_seq3*	102.09	13	Protein disulfide-isomerase P54399 *Drosophila melanogaster*	Disulfide isomerase - PDI	Ae, Cc, De, Pc, Pl, Pp
comp24941_c0_seq1	95.31	13	Platelet glycoprotein V O08742 *Mus musculus*	Leucine Rich Repeat	Ae, Pc, Pl
comp22420_c0_seq1*	90.06	16	Lipase 3 O46108 *Drosophila melanogaster*	Lipase	Ci, Lb, Ma, Nv, Ot, Ph, Pp
comp22102_c0_seq1	79.94	11	No hits found	DUF4803	Cd, Mh, Pc, Pl
comp22165_c0_seq2*	71.83	5	Lysosomal alpha-mannosidase Q8VHC8 *Cavia porcellus*	GH38 - Alpha mannosidase	De

List of proteins identified by SDS-PAGE and LC-MS/MS of crude venom extract, sorted by descending order of RPKM values, filtered for the presence of the signal peptide and highly represented in the venom gland transcriptome (i.e., in the upper third of RPKM values distribution). *Candidates selected for expression studies. Ac, *Anisopteromalus calandrae*. Ae, *Aphidius ervi*. Bh, *Bracon hebetor*. Ci, *Chelonus inanitus*. Cc, *Cotesia chilonis*. Cr, *Cotesia rubecula*. Dc, *Dinocampus coccinellae.* De*, Diversinervus elegans*. Eo*, Eupelmus orientalis*. Es, *Euplectrus separatae.* Hd*, Hyposoter didymator*. Lb, *Leptopilina boulardi*. Lh, *Leptopilina heterotoma*. Ma, *Microctonus aethiopoides*. Mh, *Microctonus hyperodae*. Nv, *Nasonia vitripennis. Ot, Ooencyrtus telenomicida. Ph, Pimpla hypochondriaca*. Pp, *Pteromalus puparum*. Pc, *Psyttalia concolor*. Pl, *Psyttalia lounsburyi*. Tb, *Tetrastichus brontispae*. Tn, *Toxoneuron nigriceps*. References for the homologs of *B. nigricans* venom proteins present in other parasitoid species are shown in Additional file 4: Table S3

### Specificity of expression in the venom gland of selected genes

**Fig. 4:**
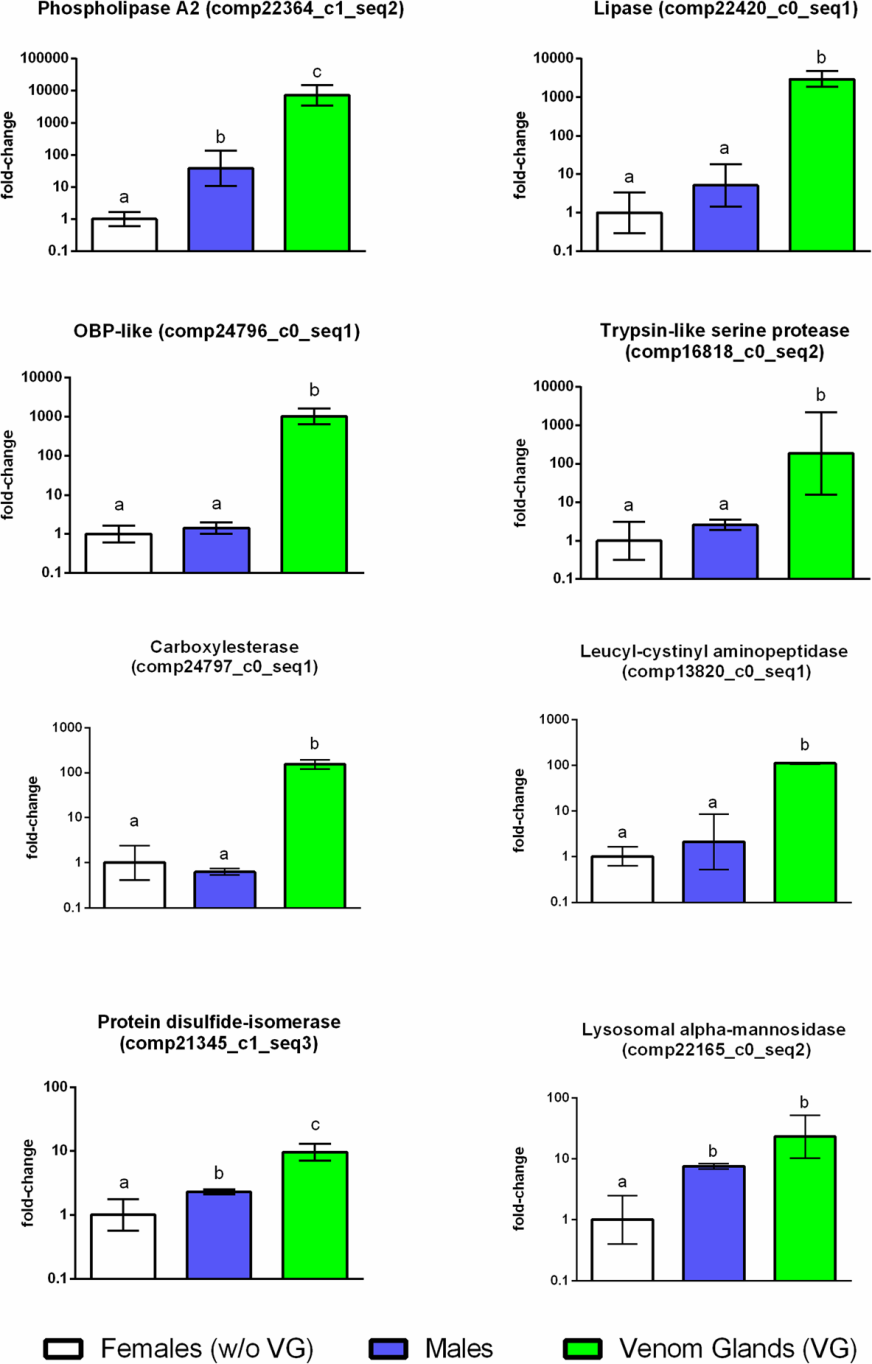
Specificity of expression in the venom glands of selected genes. Results showing the abundance of selected transcripts measured by qRT-PCR in females deprived of venom glands, males and venom glands. Results are presented as mean fold changes of three independent biological replicates, using females deprived of venom glands as calibrator. Relative expression (fold-change) is reported on Y-axis, which is plotted using a base 10 logarithmic scale. Error bars indicate standard error. Mean values denoted with different letters are significantly different (One-way ANOVA followed by Tukey’s test, *P* < 0.05)

To experimentally corroborate the validity of the approach used in the identification of the major functional players in the venom blend of *B. nigricans*, a qRT-PCR experiment was performed on total RNA extracted from venom glands, whole adult males and females deprived of the venom apparatus, focusing on a sample set of 8 genes encoding proteins selected among those showing similarity with sequences deposited in the UniProt/SwissProt database and/or highly represented in the venom gland transcriptome. Female body devoid of venom glands was used as calibrator sample. A significantly higher transcription level in venom glands was observed for all genes considered (Additional file 5: Table S4): phospholipase A2 (*P* < 0.001), lipase (*P* < 0.001), odorant binding protein (*P* < 0.001), trypsin-like serine protease (*P* < 0.001), carboxylesterase (*P* < 0.001), aminopeptidase (*P* < 0.001), protein disulfide-isomerase (*P* < 0.001) and lysosomal alpha-mannosidase (*P* < 0.005) (Fig. 4). Moreover, a much lower but significantly higher transcription level was found also in males, compared to females devoid of venom glands, for phospholipase A2 (*P* < 0.005), protein disulfide-isomerase (*P* < 0.005) and lysosomal alpha-mannosidase (*P* < 0.05) (Fig. 4).

## Discussion

Female parasitoid wasps inject complex cocktails of proteins into their hosts to induce physiological alterations which facilitate the successful development of their offspring [55]. *B. nigricans* is an ectoparasitic wasp which determines a rapid host developmental arrest by envenomation. Venom injection immobilizes the host, inhibits immune response and triggers metabolism redirection, in order to enhance host nutritional suitability for the developing parasitoid larvae [54]. To identify the major venom components involved in the host regulation, we performed an in-depth venomics approach, which combines high-throughput transcriptomics and proteomics [56].

Concurrent analysis of venom gland transcriptome and venom proteome revealed the presence of a large proportion of sequences (47.5% of the total transcripts) showing no similarity with those available in the UniProtKB database. Since parasitism factors can be very specific, displaying poor similarity among related evolutionary lineages [57–59], these unannotated proteins very likely include bioactive venom components that, however, are not easy to characterize from a functional point of view, even though they represent an untapped source of biological novelties worth of further studies.

Both transcriptome and proteome reported the presence of membrane, cytoskeletal or mitochondrial proteins, lacking the predicted signal peptide (e.g., myosin, calcium-transporting ATPase, tubulin). While these genes are expected to be found in the transcriptome of venom glands, their presence in the venom proteome very likely derives from a contamination due to cell breakage during sample collection [26].

Venom includes secreted proteins, which are expressed at different levels, and their abundance is often associated with important biological functions [15, 60]. In order to identify a robust set of proteins highly expressed in venom glands, we combined transcriptomic and proteomic data. In particular, the criteria for transcript selection were: (1) the presence of peptides identified by proteomic analysis; (2) the abundance at the transcript level; (3) the homology with known effectors in parasitoid-host interaction studies. This approach allowed the identification of a set of genes, very likely having an important role in the host regulation, which were subjected to qRT-PCR analysis, to assess the degree of specificity of their expression in the venom glands of *B. nigricans*. All these genes were highly expressed in venom glands, to a various extent, confirming their key-relevance in the venom blend.

Multiple alignments revealed that most venom proteins of *B. nigricans* are putatively functional enzymes, sharing the same conserved active sites of their homologs expressed in species lacking venom glands (Additional file 6: Figures S2-S9). Indeed, as already suggested for parasitic Hymenoptera, the mechanism of acquisition of venom components is likely related to co-option (i.e., new use of existing genes through expression alterations) rather than to duplication and neofunctionalization [61]. However, most of venom proteins identified in this study clearly diverge from homologs present both in non-venomous species, and in species using venom for defense (e.g. ants, bees) (Additional file 7: Figures S10-S17); this suggests the occurrence of a divergent evolution driven by colonization of separate host niches and specialization to different parasitic life habits.

The main components of *B. nigricans* venom are hydrolases acting on ester bonds (EC 3.1), which include a large group of different proteins, such as phospholipases A2 (PLA2) and lipases, frequently reported as venom components of several parasitoid species [55, 62, 63].

The PLA2 (*Bn*PLA2; comp22364_c1_seq2) identified by our analysis resulted highly expressed in venom glands, in accordance with the wide occurrence of this enzyme, which is one of the main venom components of Hymenoptera, including honeybees [64] and many parasitic wasps, such as *E. orientalis* [32], *Toxoneuron nigriceps* [21] and *Psyttalia concolor* [25]. *Bn*PLA2 shows 48% of identity, along 99% of the protein length, with two putative PLA2s of *B. hebetor* found by BLASTp in NCBI patented protein sequences database (GenBank: CAB42203.1; CAA03259.1). In *B. hebetor* the two sequenced PLA2s, named BrhTX-1(b) and BrhTX-1(c), are two of the four subunits of the paralyzing BrhTX-1 toxin [65]. PLA2s (EC 3.1.1.4) are a large super-family of lipolytic enzymes that cleave the glycerol backbone of phospholipids, usually in a metal-dependent reaction, to release free fatty acids and lysophospholipids [66]. These products of lipid hydrolysis (i.e., arachidonic and linoleic acid) are cytotoxic [67], as demonstrated by many studies on catalytically active venom PLA2s exerting neurotoxic [68, 69], myotoxic [70], anticoagulant [71] and antibacterial activities [72]. A pharmacological effect independent of catalytic activity is also predicted for divergent putative homologs of *Bn*PLA2 in other Hymenoptera, such as *A. compressa*, which lacks the catalytic site (H34) (Additional file 6: Figure S2 and Additional file 7: Figure S10) [73, 74].

Among transcripts highly expressed in venom glands, a lipase (*Bn*LIP; comp22420_c0_seq1) exhibits 32% of identity, along 90% of the protein length, with a homolog found in *N. vitripennis* venom (GenBank: NP_001154991.1) [75]. Lipases (EC 3.1.1.3) are also found in the venom of *Pteromalus puparum* [27], *Pimpla hypochondriaca* [76], *Chelonus inanitus* [77], *Microctonus aethiopoides* [78] and *Ooencyrtus telenomicida* [62]*.* These enzymes perform essential roles in the digestion, transport and processing of dietary lipids in most living organisms and might participate in the breakdown of the energy stores contained in the fat body of the host, in order to increase the nutritional suitability of its body fluids ingested by parasitoid larvae [79, 80]. Moreover, these enzymes may contribute by lipid hydrolysis to the generation of the toxic molecules mentioned above.

A similar function is hypothesized for another esterase, a carboxylesterase (comp24797_c0_seq1), which results highly expressed in the venom glands of *B. nigricans*. Carboxylesterases (EC 3.1.1.1) are reported as components of diverse venoms of parasitoids, including the venoms of *N. vitripennis* [75], *Hyposoter didymator* [81] and *A. calandrae* [45]. Interestingly, a putative carboxylesterase was isolated from the teratocytes of *Dinocampus coccinellae*, which are cells of embryonic origin that support the development of parasitoid larvae [79]. Considering that de novo lipid synthesis is energetically expensive [82], the mobilization and consumption of host lipids, through the action of the abovementioned lipolytic venom hydrolases, could provide a selective advantage for *B. nigricans* larvae by enhancing the host nutritional suitability.

A trypsin-like serine protease (*Bn*TRY; comp16818_c0_seq2; EC:3.4.21) is highly expressed in venom glands, compared to other tissues. *Bn*TRY shows 52% of identity, along 100% of the protein length, with a putative trypsin-like serine protease of *B. hebetor* venom found by BLASTp in NCBI patented protein sequences database (GenBank: CAB42201.1) which corresponds to “Sequence 7 from Patent WO9744355” (GenBank: A67382.1) [65]. Moreover, this protein exhibits a 23% of identity, along 90% of the sequence length, with a *N. vitripennis* venom serine protease (NP_001155042.1), which is the annotated gene in the venom glands of this pupal ectoparasitoid showing the highest expression level [83]. Serine proteases are a very common functional category in insect genomes that can have very diverse roles in parasitoid physiology. A classical function exerted by serine proteases is digestion, as occurs in the larvae of the ectoparasitoid *Euplectrus separatae*, which releases a salivary secretion containing a trypsin-like enzyme to digest the host tissues [84]. When highly expressed in venom glands, serine proteases may also play important roles in interfering with the immune response of the host by altering the proteolytic cascades activated by the detection of non-self intruders [85, 86]. Indeed, serine proteases were found in the venom of various parasitoid species, such as, for example, *Aphidius ervi* [87], *C. inanitus* [77], *N. vitripennis* [38], *P. hypochondriaca* [88], and *P. puparum* [27]. However, the disruption of the proteolytic activating cascade involved in the melanization of host hemolymph can be also induced by a mutated serine protease with inhibitory effects, such as reported for the endoparasitoid *Cotesia rubecula* [85]. Interestingly, *B. nigricans* envenomation leads to a reduced encapsulation and melanization response in the host *S. littoralis* [54], supporting the hypothesis that *Bn*TRY can be involved in the immunosuppressive syndrome.

A leucyl-cystinil aminopeptidase (*Bn*LCA; comp13820_c0_seq1; EC 3.4.11.1) shows 24% identity, along 98% of the sequence length, with an aminopeptidase N-like protein (GenBank: EFN65598.1) from *Camponotus floridanus* (Hymenoptera, Formicidae). *Bn*LCA contains a peptidase_M1 domain (pfam01433), which characterizes a family of zinc-metalloenzymes involved in the cleavage of amino acids from the amino terminus (N-terminus) of proteins or peptides [89]. Aminopeptidase activity was detected in the venom of *Cotesia chilonis* [26], *P. hypochondriaca* [90] and *Psyttalia lounsboury* [25], although no further functional data are available in the literature. On the contrary, the aminopeptidase N of *Plasmodium falciparum*, A-M1 (*Pf*A-M1) is well characterized. *Pf*A-M1 has various localizations and, in particular, in the food vacuole (a unique proteolytic organelle), where is involved in parasite metabolism and participates in the last steps of hemoglobin degradation [91]. Thus, it is reasonable to assume that *Bn*LCA may participate to the general degradation of host tissues, perhaps increasing their permeability to other venom components and facilitating nutritional exploitation of the host, as suggested for other venom zinc-metallopeptidases [92, 93].

A lysosomal alpha-mannosidase (*Bn*LAM; comp22165_c0_seq2; EC 3.2.1.24), which takes part in the sequential degradation of complex, hybrid and high-mannose N-linked oligosaccharides [94], is highly expressed in venom glands of *B. nigricans*. A homolog of *Bn*LAM has been recently identified by proteomic approach in the venom of an encyrtid endoparasitoid wasp, *Diversinervus elegans* [23]*.* However, the role of lysosomal alpha-mannosidase (LAM) and, more in general, the role of carbohydrases in entomophagous insects has been poorly characterized. LAMs, as well as the abovementioned trypsin-like serine protease and leucyl-cystinil aminopeptidase, are present in the digestive fluids of entomophagous arachnids (scorpions and spiders), which feed on their insect preys through extra-oral digestion (EOD) [95–97]. Enzymatic digestion through the action of saliva and digestive fluids represents a common strategy adopted by predatory arthropods and parasitoid wasps to exploit nutritional resources [98, 99]. In parasitic Hymenoptera, the EOD has been described in different species, as, for example, in the case of the larvae of *Trichogramma australicum* and *E. separatae* [84, 100]*,* as well as for the teratocytes of *A. ervi,* which determine the cytolytic degradation of the formed embryos of the aphid *Acyrthosiphon pisum* [101]. However, as said above, these digestive enzymes identified in the venom of *B. nigricans* can be likely involved also in a more subtle regulation of the host immunity. Indeed, it is reasonable to propose that this enzyme could induce the structural disruption of glycoconjugants which mediate the recognition of altered self domains associated with feeding wounds [102]. Then, by preventing the recruitment of hemocytes for wound healing, the feeding hole remains patent and the food uptake by wasp larvae is not impaired, as needed for all ectophagous parasitic arthropods [2].

A pheromone/general odorant binding protein (*Bn*OBP; comp24796_c0_seq1; IPR006170) results one of the most abundant components in the venom of *B. nigricans*, as suggested by the strong intensity of the corresponding band (number 27, Fig. 3) in SDS-PAGE, high RPKM (the highest in the transcriptome) and qRT-PCR data. *Bn*OBP, which has no matches in SwissProt database, shows 27% identity, along the 62% of the sequence length, with the translated transcript GECT01010095 (GenBank) from the endoparasitoid wasp *P. puparum*. OBPs participate in solubilization and transport of small hydrophobic odorant molecules and pheromones, and are characterized by six conserved cysteine residues, forming three disulfide bonds that stabilize the folded structure of the protein [103]. *Bn*OBP contains only four cysteine residues in the mature protein sequence (Additional file 6: Figure S8) and thus can be classified as Minus-C OBP (i.e., less than six cysteine residues), tough the relationship between function and structural changes for this protein family is still largely unknown [104]. It has been suggested that with less cysteine residues, Minus-C OBPs might have more structural flexibility than the Classic OBPs [105], and thus higher binding affinity for a broader number of ligands [106]. Phylogenetic reconstruction (Additional file 7: Figure S16) showed the presence of several paralogs in the venom gland transcriptome of *B. nigricans*, while few putative orthologs are found in the nr NCBI db, suggesting that OBPs are evolving through a rapid neofunctionalization [107]. The presence of members of this protein family in the venoms of parasitoid wasps has been reported for *N. vitripennis* [75], *C. inanitus* [77], *Leptopilina heterotoma* [108], *P. puparum* [109] and *A. calandrae* [45]. As hypothesized by these studies, the detection of OBPs in the venom may suggest their involvement in host selection [27, 110], when, after initial paralysis, *B. nigricans* female intensively probes the host with its ovipositor before egg laying [52]. Alternatively, beyond their canonical chemosensory role, it has been proposed that OBPs can act as carriers, mediating the solubilization of hydrophobic molecules, such as free fatty acids released by lipases [111, 112]. Then, *Bn*OBP may have a nutritional relevance for the developing parasitoid larvae acting in tandem with *Bn*LIP and, thus, contributing to lipid mobilization in the parasitized host.

A protein disulfide-isomerase (*Bn*PDI; comp21345_c1_seq3; EC 5.3.4.1) shows 73.8% identity, along the 100% of the sequence length, with a recently annotated PDI from the genome of the parasitoid wasp *Diachasma alloeum* (NCBI Reference Sequence: XP_015122546.1). This enzyme family has also been identified in the venom glands of *A. ervi* [87] and *Psytallia* species [25], as well as in the crude venom extract of *P. puparum* [27], *D. elegans* [23] and *C. chilonis* [26]. PDIs are involved in the folding of proteins by catalysing the oxidation, isomerization and reduction of disulfide bonds, that covalently link specific cysteine residues and confer stability to proteins [113, 114]. In venomous cone snails, PDIs are strictly localized in the venom glands, where they guide the folding of cysteine-rich peptide toxins (conotoxins) into their native state, while they are absent in the secreted venom [115, 116]. Indeed, PDIs are mainly localized in the endoplasmic reticulum, and their presence in the extracellular space is considered rather rare [117]. Intriguingly, *Bn*PDI sequence lacks the C-terminal motif Lys-Asp-Glu-Leu (KDEL) (Additional file 6: Figure S9), which prevents the secretion of luminal proteins from endoplasmic reticulum [118]. Therefore, the PDI presence in the venom of *B. nigricans*, if not due to the breakage of venom glands during venom collection, could represent a new interesting case of secretion, worth of further studies.

## Conclusions

The integration of transcriptomics and proteomics used in the present work provides the first description of the main components of *B. nigricans* venom and contributes to the expansion of the limited information available for the venom of ectophagous parasitoids, that permanently paralyze and rapidly suppress their victims (i.e., idiobiont parasitoids). The most abundant venom components of *B. nigricans*, mainly esterases and proteases likely involved in paralysis (*Bn*PLA2) and digestion of the host’s tissues, are in line with a typical ectoparasitic idiobiont life-style. Enzymes with lipolytic activity are likely involved in the mobilization of storage nutrients from fat bodies and/or in the release of neurotoxic fatty acids inducing paralysis, while enzymes related to carbohydrate catabolism could be responsible for the alteration of glycoconjugants which mediate the recognition of altered self domains mediating wound healing, and may induce the observed changes of the carbohydrate titer in the host hemolymph [54]. The host regulation strategy emerging from this latter study is well corroborated by the discovery of venom component that can have immunosuppressive activity, such as a trypsin-like serine protease (*Bn*TRY) and a lysosomal alpha-mannosidase (*Bn*LAM).

The venom gland transcriptome of *B. nigricans* shows the occurrence only of a few transcripts in common with *B. hebetor* [47]. The most significant similarity is the presence in both species of neurotoxic molecules (i.e., phospholipases), which were previously identified in *B. hebetor* and patented [65]; only a few other transcripts are shared (arginine kinase and venom acid phosphatase). Even though the data available for *B. hebetor* are somewhat limited, it is quite evident that these two closely related species, attacking the same group of insect hosts, rely upon a molecular toolkit for host regulation and exploitation which is only partly conserved, suggesting the occurrence of an ancestral broad molecular biodiversity of the venom blend, which has been one of the major pre-requisites allowing the evolutionary diversification of parasitic Hymenoptera.

The identification of these venom components can have important implications from an applied perspective, given their bioinsecticide activity [119]. Indeed, the functional characterization of the venom proteins identified in the present study will offer new tools to develop bioinspired strategies for pest control, based on the use of natural antagonists beyond the organism level, as a source of insecticide molecules.

## Methods

### Parasitoid and host rearing

*B. nigricans* was reared on larvae of the noctuid moth *S. littoralis,* which were used as laboratory host, maintained on artificial diet as previously described [120]. Adults of *B. nigricans* were maintained at 27 ± 1 °C, 70 ± 5% RH and 16:8 h (L:D) photoperiod, as previously described [54]. The starting material of *B. nigricans* colony, used for transcriptomic and proteomic studies, was kindly donated by Lucia Zappalà (University of Catania, Catania, Italy) and derived from a laboratory population established in 2010 with specimens collected in Italy, Sicily region (Catania area), to which field material from the same area was added every year to refresh the colony. *S. littoralis* colony was established in 2010 and refreshed with insects provided by ISAGRO (Novara, Italy).

### Venom gland collection and RNA isolation

Female wasps were anaesthetized on ice and dissected in sterile PBS (Phosphate Buffered Saline: 137 mM NaCl, 2.7 mM KCl, 10 mM phosphate buffer, pH 7.4) by grasping the ovipositor tip with fine forceps, under a stereoscope (Discovery v8, Zeiss). Reservoir and venom glands (Fig. 1) were separated using dissection needles and forceps. Venom glands were collected into a microcentrifuge tube containing 300 μl of TRIzol® Reagent (Thermo Fisher Scientific, Waltham, MA, USA) kept on ice and, then, stored at − 80 °C until RNA extraction. Total RNA extraction was performed according to the manufacturer’s directions, using TRIzol® Reagent, while the PureLink™ RNA Micro Kit was used to purify and concentrate RNA (Thermo Fisher Scientific). In order to eliminate genomic DNA, an on-column DNase treatment (PureLink®DNase, Thermo Fisher Scientific) was carried out. An amount of 2.4 μg of total RNA was obtained from venom glands of 80 female wasps. The quantity and the quality of total RNA were assessed using Varioskan Flash spectrophotometer (Thermo Fisher Scientific) and, prior to cDNA library construction, with Agilent 2100 Bioanalyzer (Agilent Technologies, Santa Clara, CA, USA).

### cDNA library construction, sequencing and computational analysis

TruSeq (Illumina) stranded cDNA sequencing libraries were constructed. The 100 bp paired-end sequencing run was performed on the Illumina HiSeq1500 platform. Raw sequences were trimmed from adapters using Cutadapt [121]. The remaining sequences were checked for quality (Q20 and error rate 0.01) using Trim Galore v. 0.4.0 [122, 123]. The de novo transcriptome assembly of the cleaned reads was performed with Trinity, setting the k-mer length at the default value (25) [124, 125]. To remove redundancy, the assembled transcripts were further collapsed using CD-HIT-EST, setting identity (−c option) and coverage (−s option) equal to 1 [126]. The sequences obtained were annotated by BLASTx comparisons versus the UniProtKB/SwissProt protein database [127], using default parameters and setting the e-value threshold at 1e-5. GO terms assignments, encoded enzymes and functional domain identifications were supplemented to the functional annotation by the web based pipeline FastAnnotator [128]. Signal peptides for translated transcripts were predicted using SignalP 4.0 software [129].

In order to detect the presence of putative toxins in the resulting data, the protein hits associated with each transcript by the BLASTx search versus the UniProtKB database were crossed with the sequences deposited in the UniProtKB/Swiss-Prot Tox-Prot db (http://www.uniprot.org/program/Toxins), which are categorized in toxin and venom proteins [130]. The assembled and annotated venom gland transcriptome was used to generate a custom-made protein database, by translating the six reading frames of the nucleotide sequences in their corresponding amino acid sequences by SEQtools (http://www.seqtools.dk/). The relative expression level of each transcript was estimated calculating the RPKM (Reads per Kilobase per Million) based on the number of reads re-mapped on the assembled sequences using Bowtie2 alignment tool [131].

### Extraction and SDS-PAGE fractionation of venom proteins

Adult females of *B. nigricans* were dissected in ice-cold Pringle’s solution [132] with 1 mM phenylmethylsulfonyl fluoride, as described above. After resection from venom glands, the reservoir was gently ruptured, using dissecting needles, and the exuding crude venom content transferred to a microcentrifuge tube. The crude extract was centrifuged at 5000×g, at 4 °C, for 5 min to remove cell debris, and the supernatant was thus filtered through a 0.22 μm filter (Millipore, Merck KGaA, Germany) and stored at − 80 °C until use. Protein concentration of the venom extracted was assessed by Bradford method [133].

In total, 12 μg of venom proteins were fractioned on 10% SDS-PAGE minigel and stained with colloidal Coomassie (0.1% (w/v) Coomassie Brilliant Blue G250 (Thermo Fisher Scientific), 2% (w/v) ortophosphoric acid, 10% (w/v) ammonium sulfate). Selected protein bands were thus excised from the gel and washed in a solution of 50 mM ammonium bicarbonate (pH 8.0) in 50% acetonitrile until complete destaining. Each gel slice was resuspended in 50 mM ammonium bicarbonate and incubated with 100 ng of trypsin for 2 h at 4 °C and overnight at 37 °C. The supernatant containing the resulting mixture of peptides was removed and the gel pieces were re-extracted with acetonitrile. The two fractions were then pooled and freeze-dried.

### LC/MS-MS and protein identification

The peptide mixture from each gel slice was analyzed by LC/MS-MS, using the LC/MSD Trap XCT Ultra (Agilent Technologies, Palo Alto, CA), equipped with a 1100 HPLC system and a chip cube (Agilent Technologies). After loading, the peptide mixture (8 μl in 0.2% (v/v) formic acid in water) was first concentrated and washed at 4 μl/min in a 40 nl enrichment column (Agilent Technologies chip), with 0.1% formic acid as eluent (eluent A). The sample was then fractionated on a C18 reverse-phase capillary column (75 μm × 43 mm in the Agilent Technologies chip) at a flow rate of 300 nl/min, with a linear gradient of eluent B (0.1% (v/v) formic acid in acetonitrile) in A, from 5 to 60% in 50 min. Elution was monitored with the mass spectrometers without any splitting device. Peptide analysis was performed using data-dependent acquisition of one MS scan (m/z range from 400 to 1600 Da/e), followed by MS/MS scans of the three most abundant ions in each MS scan. Dynamic exclusion was used to acquire a more complete survey of the peptides by automatic recognition and temporary exclusion (2 min) of ions from which definitive mass spectral data had previously been acquired. Moreover, a permanent exclusion list of the most frequent peptide contaminants (keratins and trypsin peptides) was included in the acquisition method in order to focus the analyses on significant data.

The resulting MS/MS spectra were searched against the translated *B. nigriceps* transcriptome with Mascot 2.4 (Matrix Science, Boston, MA, USA), using the Ion Search option. Carbamidomethyl of cysteine was set as fixed modification, while Pyro-Carbamidomethyl of cysteine (N-term C), Pyro-Glu (N-term Q) and oxidation of methionine were set as variable modifications. The other parameters were set at 0.6 Da for mass tolerance, ± 660 ppm for precursor ion tolerance and 1 for maximum missed cleavages allowed.

### Quantitative RT-PCR (qRT-PCR) analysis

To confirm in vivo the result of proteomic and transcriptomic analyses, the expression profiles of selected genes were assessed using qRT-PCR. Total RNA was separately extracted from (1) venom glands, (2) females deprived of the venom glands and (3) males, using TRIzol® reagent (Thermo Fisher Scientific) according to the manufacturer’s protocol. Each sample was formed by a pool of RNA obtained from 5 individuals and was analyzed in triplicate on a Step One Real Time PCR System (Applied Biosystems, Carlsbad, CA, USA). Differential relative expression of the target genes was measured by one-step qRT-PCR, using the SYBR Green PCR Kit (Applied Biosystems), according to the manufacturer’s instructions. *B. nigricans* 40S ribosomal protein S3 (RPS3) gene (GenBank accession number: MK631956) was used as endogenous control for RNA loading. All primers were designed using Primer Express, version 1.0 software (Applied Biosystems) and are reported in (Additional file 8: Table S5). Relative gene expression data were analyzed using the ∆∆Ct method [134, 135]. For validation of the ∆∆Ct method, the difference between the Ct value of the target and the Ct value of *RPS3* transcripts [∆Ct = Ct(target gene)-Ct (*RPS3*)] was plotted versus the log of ten-fold serial dilutions (100, 10, 1, 0.1 and 0.01 ng) of the purified RNA samples. The plot of log total RNA input versus ∆Ct displayed a slope less than 0.1, indicating that the efficiencies of the two amplicons were approximately equal. The relative expression of the target genes in female body deprived of the venom glands was used as calibrator (relative expression = 1). The results are presented as mean fold changes of three independent biological replicates. ∆Cts were compared using one-way analysis of variance (ANOVA) and Tukey’s test with statistical significance set at *P* < 0.05. All statistical analyses were performed using the Statistical Analysis Systems software (Sigma Stat Statistical Software, SPSS Science, Chicago, IL, USA).

### Sequence analysis and phylogeny reconstruction

Putative homologous sequences of the most representative venom proteins were identified by sequence similarity searches through a BlastP analysis versus the non-redundant NCBI database (nr NCBI, release October 2019) and Swiss-Prot (release 2019_10 of 13-Nov-19). Additional rounds of BLASTp analyses were performed selecting Arachnida (taxid:6854) and Hymenoptera (taxid:7399) groups in the nr NCBI collection. However, using the *Bn*OBP protein sequence as a query resulted in only 3 hits below E-value threshold of 10^− 1^. Therefore a tBLASTn search against the transcriptome of *B. nigricans* venom glands was also performed. Most representative hits selected below the E-value threshold of 10^− 5^ were aligned using Muscle 3.8 [136], with default settings.

The alignments of venomous and non-venomous orthologs were plotted using Jalview 2 [137], and sequences were analyzed with ScanProsite [138] and HMMscan (HmmerWeb version 2.40.0) tools, in order to identify active sites and conserved patterns.

To reconstruct phylogeny, alignments were manually trimmed to avoid comparisons of non-conserved regions present only in a subset of the taxa. Best-fit model of amino acid substitution and phylogenetic reconstruction was performed using RAxML 8.2.12 [139]. The maximum-likelihood tree was run for 1000 bootstrap replicates and the tree figure was plotted using FigTree v1.4.3.

## Supplementary information

**Additional file 1: Figure S1.** Distribution of transcripts abundance expressed in RPKM

**Additional file 2: Table S1.** List of the most abundant (RPKM>100) annotated transcripts encoding putatively secreted proteins (i.e., positive to SignalP analysis)

**Additional file 3: Table S2.** Venom proteome annotated by BLAST search

**Additional file 4: Table S3.** Homologs of *B. nigricans* venom proteins occurring in other parasitoid species

**Additional file 5: Table S4.** One-way ANOVA of ΔCt values recorded in venom glands, females devoid of venom glands and males

**Additional file 6: Figures S2-S9.** Multiple sequence alignments of most representative *B. nigricans* venom proteins

**Additional file 7: Figures S10-S17.** Phylogenetic trees of most representative *B. nigricans* venom proteins

**Additional file 8: Table S5.** Primers used for qRT-PCR analysis of selected venom components

## Data Availability

The raw sequences have been deposited at SRA-NCBI (Accession Number: SRR9041613). Further supplementary data are provided in the Additional files 1-8.

## Abbreviations

ANOVA: Analysis of Variance
ATPase: Adenosine triphosphatase
BLAST: Basic Local Alignment Search Tool
*Bn*LAM: *Bracon nigricans* lysosomal alpha-mannosidase
*Bn*LCA: *Bracon nigricans* leucyl-cystinil aminopeptidase
*Bn*LIP: *Bracon nigricans* lipase
*Bn*OBP: *Bracon nigricans* odorant binding protein
*Bn*PDI: *Bracon nigricans* protein disulfide-isomerase
*Bn*PLA2: *Bracon nigricans* phospholipase A2
*Bn*TRY: *Bracon nigricans* trypsin-like serine protease
cDNA: Complementary DNA
Ct: Cycle threshold
DNAse: Deoxyribonuclease
EOD: Extra-oral digestion
GABA: Gamma-aminobutyric acid
GO: Gene Ontology
HPLC: High Performance Liquid Chromatography
LAM: Lysosomal alpha-mannosidase
LC/MSD: Liquid Chromatography/Mass Selective Detector
LC/MS-MS: Liquid Chromatography coupled with tandem Mass Spectrometry
LCA: Leucyl-cystinil aminopeptidase
LIP: Lipase
MS: Mass spectrometry
OBP: Odorant binding protein
PDI: Protein disulfide-isomerase
*Pf*A-M1: *Plasmodium falciparum* aminopeptidase N
PLA2: Phospholipase A2
qRT-PCR: Quantitative Reverse Transcriptase - Polymerase Chain Reaction
RPKM: Reads Per Kilobase per Million
RPS3: Ribosomal Protein S3
SDS-PAGE: Sodium Dodecyl Sulphate - PolyAcrylamide Gel Electrophoresis
TRY: Trypsin-like serine protease
UniProtKB: UniProt Knowledgebase
