# Supplementary material for: Venomics of the ectoparasitoid wasp Bracon nigricans
Source: BMC Genomics. 2020 Jan 10;21:34. doi: 10.1186/s12864-019-6396-4 (PMC6954513; doi:10.1186/s12864-019-6396-4)
Supplement: Supplementary file 1 — Additional file 1: Figure S1. Distribution of transcripts abundance expressed in RPKM [file 12864_2019_6396_MOESM1_ESM.docx]

**Fig. S1. Distribution of transcript abundance expressed in RPKM.** Histograms reporting the number of transcripts (Y- axis) per RPKM range (i.e. a measure of abundance within the transcriptome). Seven classes of abundance are reported, from low (in blue) to high (in black) RPKM values.

**
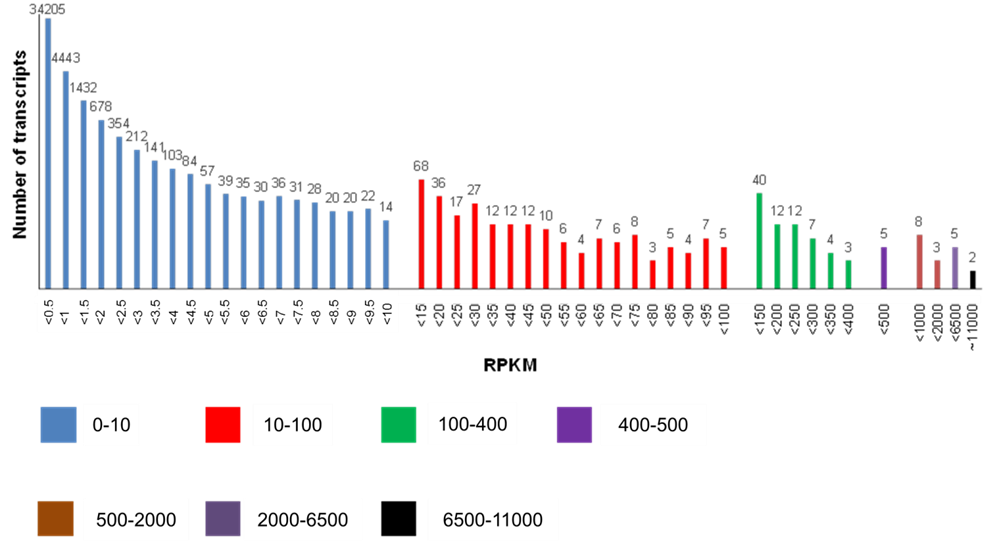
**
