# Supplementary material for: Venomics of the ectoparasitoid wasp Bracon nigricans
Source: BMC Genomics. 2020 Jan 10;21:34. doi: 10.1186/s12864-019-6396-4 (PMC6954513; doi:10.1186/s12864-019-6396-4)
Supplement: Supplementary file 2 — Additional file 2: Table S1. List of the most abundant (RPKM>100) annotated transcripts encoding putatively secreted proteins (i.e., positive to SignalP analysis) [file 12864_2019_6396_MOESM2_ESM.docx]

**Table S1. List of the most abundant (RPKM>100) annotated transcripts encoding putatively secreted proteins (i.e., positive to SignalP analysis).**

| Transcript ID | RPKM | Best match in UniProtKB db | SDS-PAGE band number |
| --- | --- | --- | --- |
| comp22364_c1_seq2 | 9.209,46 | Phospholipase A2 isozymes PA3A/PA3B/PA5 OS=*Heloderma suspectum* PE=1 SV=3 | 26 |
| comp24799_c0_seq1 | 4.703,32 | Venom metalloproteinase 2 OS=*Eulophus pennicornis* PE=2 SV=1 |  |
| comp24801_c0_seq1 | 4.258,98 | Phospholipase A2 OS=*Bombus pensylvanicus* PE=1 SV=1 |  |
| comp24797_c0_seq1 | 3.996,50 | Venom carboxylesterase-6 OS=*Apis mellifera* PE=2 SV=1 | 11 |
| comp24806_c0_seq1 | 3.088,82 | Venom allergen 5 OS=*Vespa magnifica* PE=1 SV=2 | 23 |
| comp24804_c0_seq1 | 2.976,88 | Chymotrypsin-like elastase family member 2A OS=*Rattus norvegicus* GN=Cela2a PE=2 SV=1 |  |
| comp24795_c0_seq1 | 2.913,22 | Venom metalloproteinase 3 OS=*Eulophus pennicornis* PE=2 SV=1 |  |
| comp17537_c0_seq1 | 2.733,44 | Phospholipase A2 OS=*Apis cerana cerana* PE=2 SV=1 |  |
| comp24411_c0_seq1 | 2.029,31 | A disintegrin and metalloproteinase with thrombospondin motifs 12 OS=*Homo sapiens* GN=ADAMTS12 PE=1 SV=2 |  |
| comp24841_c0_seq1 | 1.486,90 | 40S ribosomal protein S15 OS=*Elaeis oleifera* GN=RPS15 PE=2 SV=1 |  |
| comp24830_c0_seq1 | 1.216,56 | Trypsin epsilon OS=*Drosophila erecta* GN=epsilonTry PE=3 SV=1 |  |
| comp24892_c0_seq1 | 687,86 | Acidic phospholipase A2 PA4 OS=*Heloderma suspectum* PE=1 SV=2 |  |
| comp20928_c0_seq1 | 419,72 | Serine protease easter OS=*Drosophila melanogaster* GN=ea PE=1 SV=3 |  |
| comp14255_c0_seq1 | 416,55 | Chymotrypsin-like elastase family member 2A OS=*Homo sapiens* GN=CELA2A PE=1 SV=1 |  |
| comp16667_c0_seq1 | 411,02 | Kallikrein 1-related peptidase b9 OS=*Mus musculus* GN=Klk1b9 PE=2 SV=1 |  |
| comp24144_c1_seq2 | 400,13 | Venom acid phosphatase Acph-1 OS=*Apis mellifera* PE=1 SV=1 |  |
| comp13763_c0_seq1 | 375,73 | Defensin-1 OS=*Apis mellifera* PE=1 SV=3 |  |
| comp24144_c1_seq1 | 367,17 | Venom acid phosphatase Acph-1 OS=*Apis mellifera* PE=1 SV=1 |  |
| comp18212_c0_seq1 | 302,69 | Leukocyte elastase inhibitor OS=*Sus scrofa* GN=SERPINB1 PE=1 SV=1 |  |
| comp23102_c0_seq1 | 293,01 | Peptidyl-prolyl cis-trans isomerase B OS=*Gallus gallus* GN=PPIB PE=2 SV=1 |  |
| comp16818_c0_seq2 | 288,36 | Chymotrypsin-2 OS=*Anopheles gambiae* GN=CHYM2 PE=2 SV=3 | 20 |
| comp23128_c0_seq5 | 269,76 | Mast cell protease 4 OS=*Rattus norvegicus* GN=Mcpt4 PE=2 SV=1 |  |
| comp15164_c0_seq1 | 267,16 | Venom allergen 3 OS=*Solenopsis invicta* PE=1 SV=2 |  |
| comp24903_c0_seq1 | 259,46 | Protein yellow OS=*Drosophila yakuba* GN=y PE=3 SV=1 |  |
| comp23128_c0_seq1 | 251,09 | Mast cell protease 2 OS=*Rattus norvegicus* GN=Mcpt2 PE=1 SV=1 |  |
| comp24931_c0_seq1 | 213,75 | Ferritin light chain OS=*Oryctolagus cuniculus* GN=FTL PE=2 SV=2 |  |
| comp24949_c0_seq1 | 197,16 | Hymenoptaecin OS=*Apis mellifera* PE=2 SV=1 |  |
| comp24926_c0_seq1 | 194,41 | General odorant-binding protein 56a OS=*Drosophila melanogaster* GN=Obp56a PE=1 SV=1 |  |
| comp13820_c0_seq1 | 156,34 | Aminopeptidase M1-A OS=*Oryza sativa* subsp. *japonica* GN=Os02g0218200 PE=2 SV=1 | 6,7 |
| comp15122_c0_seq1 | 147,89 | Cathepsin L OS=*Sarcophaga peregrina* PE=1 SV=1 |  |
| comp21267_c0_seq1 | 144,4 | Leukocyte elastase inhibitor C OS=*Mus musculus* GN=Serpinb1c PE=2 SV=1 |  |
| comp13733_c0_seq1 | 138,58 | Venom acid phosphatase Acph-1 OS=*Apis mellifera* PE=1 SV=1 |  |
| comp13733_c0_seq2 | 137,48 | Venom acid phosphatase Acph-1 OS=*Apis mellifera* PE=1 SV=1 |  |
| comp20910_c0_seq4 | 133,49 | Granzyme M OS=*Homo sapiens* GN=GZMM PE=1 SV=2 |  |
| comp20910_c0_seq1 | 131,6 | Granzyme M OS=*Homo sapiens* GN=GZMM PE=1 SV=2 |  |
| comp20910_c0_seq3 | 125,94 | Granzyme M OS=*Homo sapiens* GN=GZMM PE=1 SV=2 |  |
| comp20910_c0_seq2 | 124,19 | Granzyme M OS=*Homo sapiens* GN=GZMM PE=1 SV=2 |  |
| comp24217_c0_seq1 | 123,27 | Maltase 1 OS=*Drosophila virilis* GN=Mal-B1 PE=3 SV=2 |  |
| comp17079_c0_seq1 | 114,77 | CHH-like protein OS=*Bombyx mori* GN=CHHL PE=2 SV=1 |  |
| comp14609_c0_seq1 | 112,27 | Serine protease inhibitor I/II OS=*Schistocerca gregaria* PE=1 SV=1 |  |
| comp24189_c0_seq1 | 111,82 | Vitellogenin OS=*Apis mellifera* GN=Vg PE=1 SV=1 |  |
| comp24969_c0_seq1 | 111,5 | Peptidoglycan recognition protein OS=*Bombyx mori* PE=1 SV=1 |  |
| comp20901_c0_seq2 | 107,24 | Alaserpin OS=*Manduca sexta* PE=1 SV=1 |  |
| comp24976_c0_seq1 | 104,98 | Probable salivary secreted peptide OS=*Bombus ignitus* PE=2 SV=1 |  |
| comp24284_c0_seq1 | 102,21 | Tyramine beta-hydroxylase OS=*Drosophila melanogaster* GN=Tbh PE=1 SV=1 |  |
| comp21289_c0_seq1 | 102,18 | Retinoid-inducible serine carboxypeptidase OS=*Rattus norvegicus* GN=Scpep1 PE=2 SV=1 |  |
| comp21345_c1_seq3 | 102,09 | Protein disulfide-isomerase OS=*Drosophila melanogaster* GN=Pdi PE=2 SV=1 | 13 |
| comp21296_c0_seq1 | 101,38 | Podocan-like protein 1 OS=*Mus musculus* GN=Podnl1 PE=2 SV=2 |  |
