# Supplementary material for: Venomics of the ectoparasitoid wasp Bracon nigricans
Source: BMC Genomics. 2020 Jan 10;21:34. doi: 10.1186/s12864-019-6396-4 (PMC6954513; doi:10.1186/s12864-019-6396-4)
Supplement: Supplementary file 3 — Additional file 3: Table S2. Venom proteome annotated by BLAST search [file 12864_2019_6396_MOESM3_ESM.docx]

**Table S2. Venom proteome annotated by BLAST search.**

| Transcript ID | SwissProtdb | UniProtKBdb | Pep | Score |  |
| --- | --- | --- | --- | --- | --- |
| **Band 1** |  |  |  |  |  |
| comp24426_c0_seq2 | Myosin heavy chain  P05661 *Drosophila* | K7IRT4*Nasoniavitripennis* | 39 | 2135 |  |
| comp23954_c0_seq2 | Ca-transp ATPase sarc/ER type  P22700 *Drosophila* | K7IPT6*Nasoniavitripennis* | 22 | 1161 |  |
| comp18007_c0_seq2 | Paramyosin, long form  P35415 *Drosophila* | K7J7N4*Nasoniavitripennis* | 8 | 269 |  |
| comp22102_c0_seq1 | No hitsfound |  | 4 | 141 |  |
| **Band 2** |  |  |  |  |  |
| comp24426_c0_seq4 | Myosin heavy chain  P05661 *Drosophila* | K7IRT4*Nasoniavitripennis* | 61 | 3693 |  |
| comp24964_c0_seq1 | Apolipophorins  Q9U943 *Locusta migratoria* | E2A7K7*Camponotusfloridanus* | 7 | 375 |  |
| comp33033_c0_seq1 | Apolipophorins  Q9U943 *Locusta migratoria* | E2A7K7*Camponotusfloridanus* | 3 | 153 |  |
| **Band 3** |  |  |  |  |  |
| comp24308_c0_seq1 | Vitellogenin-6  Q94637 *Oscheiustipulae* | E2C964*Harpegnathos saltator* | 22 | 1086 |  |
| comp23954_c0_seq1 | Ca-transp ATPase sarc/ER type  Q7PPA5 *Anopheles gambiae* | K7IPT6*Nasoniavitripennis* | 7 | 471 |  |
| comp24673_c0_seq1 | Clathrin heavy chain  P29742 *Drosophila* | V9IKI6*Apiscerana* | 7 | 284 |  |
| comp18379_c0_seq1 | Arginine kinase  O61367 *Apis mellifera* | G3FP78*Bombushypocrita* | 4 | 194 |  |
| comp22460_c0_seq1 | Pyruvatekinase  O62619 *Drosophila* | E2AJ75*Camponotusfloridanus* | 4 | 177 |  |
| **Band 4** |  |  |  |  |  |
| comp24426_c0_seq2 | Myosin heavy chain P05661 *Drosophila* | K7IRT4*Nasoniavitripennis* | 57 | 3026 |  |
| comp23954_c0_seq2 | Ca-transp ATPase sarc/ER type  P22700 *Drosophila* | K7IPT6*Nasoniavitripennis* | 17 | 919 |  |
| comp18379_c0_seq2 | Arginine kinase  O61367 *Apis mellifera* | G3FP78*Bombushypocrita* | 4 | 218 |  |
| comp26660_c0_seq1 | Hypoxia up-regulated protein 1  Q566I3*Xenopuslaevis* | E2A725*Camponotusfloridanus* | 4 | 205 |  |
| **Band 5** |  |  |  |  |  |
| comp24426_c0_seq2 | Myosin heavy chain  P05661 *Drosophila* | K7IRT4*Nasoniavitripennis* | 19 | 956 |  |
| comp21536_c0_seq1 | Pyruvate carboxylase  Q05920 *Musmusculus* | E2AWL4*Camponotusfloridanus* | 12 | 689 |  |
| comp23954_c0_seq1 | Ca-transp ATPase sarc/ER type  Q7PPA5 *Anopheles gambiae* | K7IPT6*Nasoniavitripennis* | 9 | 519 |  |
| comp25466_c0_seq1 | Ubq-like modifier-activEnz 1  P22314 *Homo sapiens* | E2C890*Harpegnathos saltator* | 10 | 440 |  |
| comp18379_c0_seq1 | Arginine kinase  O61367 *Apis mellifera* | E1U7W2*Pteromaluspuparum* | 7 | 355 |  |
| comp27249_c0_seq1 | Aminopeptidase N  P91887*Plutellaxylostella* | E2BB81*Harpegnathos saltator* | 6 | 298 |  |
| comp22165_c0_seq2 | Lysosomal alpha-mannosidase  Q8VHC8 *Cavia porcellus* | K7J2I6*Nasoniavitripennis* | 7 | 302 |  |
| comp12616_c0_seq1 | Thyrotropin-releasing Horm-degr Enzyme  Q9UKU6 *Homo sapiens* | K7INE4*Nasoniavitripennis* | 4 | 264 |  |
| **Band 6** |  |  |  |  |  |
| comp18007_c0_seq2 | Paramyosin, long form  P35415 *Drosophila* | K7J7N4*Nasoniavitripennis* | 36 | 1946 |  |
| comp23954_c0_seq1 | Ca-transp ATPase sarc/ER type  Q7PPA5 *Anophelesgambiae* | K7IPT6*Nasoniavitripennis* | 32 | 1742 |  |
| comp23143_c0_seq2 | 2-oxoglutarate dehydrogenase  Q148N0*Bostaurus* | F4X6J5*Acromyrmexechinatior* | 30 | 1397 |  |
| comp13820_c0_seq1 | Leucyl-cystinylaminopeptidase  P97629 *Rattusnorvegicus* | E2BB85*Harpegnathos saltator* | 17 | 922 |  |
| comp23948_c0_seq1 | Vinculin  O46037 *Drosophila* | E2BTN6*Harpegnathos saltator* | 8 | 491 |  |
| comp20972_c0_seq1 | Presequenceprotease  Q5JRX3 *Homo Sapiens* | E9IPF6*Solenopsisinvicta* | 10 | 438 |  |
| comp18242_c0_seq1 | 97 kDa heat shock protein  Q06068 *Strongylocentrotuspurpuratus* | S4PX99*Parargeaegeria* | 7 | 317 |  |
| comp24713_c0_seq1 | Staphylococcal nuclease domain-cont P 1  Q863B3*Bostaurus* | V9IGS7*Apiscerana* | 5 | 246 |  |
| **Band 7** |  |  |  |  |  |
| comp17149_c0_seq1 | Glutamylaminopeptidase  Q07075 *Homo Sapiens* | E2BB85*Harpegnathos saltator* | 24 | 1384 |  |
| comp18007_c0_seq2 | Paramyosin, long form  P35415 *Drosophila* | K7J7N4*Nasoniavitripennis* | 20 | 1219 |  |
| comp24009_c1_seq3 | Alpha-actinin, sarcomeric  P18091 *Drosophila* | E2ACU8*Camponotusfloridanus* | 25 | 1202 |  |
| comp23954_c0_seq1 | Ca-transp ATPase sarc/ER type  Q7PPA5 *Anophelesgambiae* | K7IPT6*Nasoniavitripennis* | 18 | 1008 |  |
| comp24430_c0_seq1 | Endoplasmin  Q95M18*Bostaurus* | E2AMV4*Camponotusfloridanus* | 14 | 784 |  |
| comp22940_c0_seq2 | Filamin-A  Q9VEN1*Drosophila* | E2BBG1*Harpegnathos saltator* | 16 | 781 |  |
| comp23143_c0_seq1 | 2-oxoglutarate dehydrogenase  Q60HE2 *Macaca fascicularis* | E2AHW3*Camponotusfloridanus* | 14 | 682 |  |
| comp13820_c0_seq1 | Leucyl-cystinylaminopeptidase  P97629 *Rattusnorvegicus* | E2BB85*Harpegnathos saltator* | 10 | 586 |  |
| comp26029_c0_seq1 | Glycogen phosphorylase  Q9XTL9 *Drosophila* | E2BWP7*Harpegnathossaltator* | 12 | 574 |  |
| comp24426_c0_seq3 | Myosin heavy chain  P05661 *Drosophila* | K7IRT4*Nasoniavitripennis* | 9 | 432 |  |
| comp22531_c0_seq2 | Glycogen phosphorylase  Q9XTL9 *Drosophila* | E2BWP7*Harpegnathossaltator* | 9 | 378 |  |
| comp21027_c0_seq1 | Puromycin-sensitive aminopeptidase  Q11011*Musmusculus* | V9IL44*Apiscerana* | 5 | 284 |  |
| **Band 8** |  |  |  |  |  |
| comp23136_c0_seq1 | Aconitatehydratase  Q99KI0 *Musmusculus* | E2BSG2*Harpegnathos saltator* | 28 | 1452 |  |
| comp18078_c0_seq1 | No hitsfound | A9YME0*Microctonushyperodae* | 18 | 904 |  |
| comp14282_c0_seq1 | No hitsfound | A9YME0*Microctonushyperodae* | 17 | 748 |  |
| **Band 9** |  |  |  |  |  |
| comp24894_c0_seq1 | No hitsfound | E1ZZB9*Camponotusfloridanus* | 24 | 1302 |  |
| comp24599_c1_seq1 | Endothelin-converEnzyme-like 1  O95672 *Homo sapiens* | W8BVC8*Ceratitis capitata* | 20 | 999 |  |
| comp23895_c0_seq1 | Endothelin-convertingEnzyme 1  P42892 *Homo sapiens* | K7JA72*Nasoniavitripennis* | 21 | 954 |  |
| comp24555_c1_seq1 | Moesin/ezrin/radixinhomolog 1  B0WYY2 *Culexquinquefasciatus* | E2C7G4*Harpegnathos saltator* | 15 | 791 |  |
| comp18045_c0_seq1 | Heat shock 70 kDa P cognate 3  P29844 *Drosophila* | E2AY69*Camponotusfloridanus* | 11 | 658 |  |
| comp24916_c0_seq1 | No hitsfound | A9YME0*Microctonushyperodae* | 12 | 622 |  |
| comp23518_c0_seq1 | Dipeptidylpeptidase 3  Q9VHR8 *Drosophila* | E2B1G2*Camponotusfloridanus* | 11 | 463 |  |
| comp23136_c0_seq1 | Aconitatehydratase  Q99KI0 *Musmusculus* | E2BSG2*Harpegnathos saltator* | 7 | 366 |  |
| comp20936_c0_seq1 | No hitsfound | E9IV56*Solenopsisinvicta* | 7 | 354 |  |
| comp14043_c0_seq1 | No hitsfound | E6ZCL5*Chelonusinanitus* | 7 | 311 |  |
| comp17149_c0_seq1 | Glutamylaminopeptidase  Q07075 *Homo sapiens* | E2BB80*Harpegnathos saltator* | 4 | 315 |  |
| comp23189_c0_seq1 | No hitsfound | E1ZZB9*Camponotusfloridanus* | 6 | 278 |  |
| comp24195_c0_seq2 | Calpain-B  Q9VT65 *Drosophila* | V9I6P7*Apiscerana* | 6 | 279 |  |
| **Band 10** |  |  |  |  |  |
| comp24842_c0_seq1 | No hitsfound | E2B3J2*Harpegnathossaltator* | 24 | 1235 |  |
| comp20898_c0_seq1 | No hitsfound | E2B3J2*Harpegnathos saltator* | 20 | 1127 |  |
| comp24912_c0_seq1 | Heat shock 70 kDa P cognate 4  Q9U639 *Manducasexta* | K7IQ77*Nasoniavitripennis* | 19 | 1056 |  |
| comp23743_c2_seq1 | V-type proton ATPase catal. sub. A  Q2TJ56 *Aedesalbopictus* | K7INR1*Nasoniavitripennis* | 20 | 971 |  |
| comp18045_c0_seq1 | Heat shock 70 kDa protein cognate 3  P29844 *Drosophila* | K7ITP5*Nasoniavitripennis* | 14 | 827 |  |
| comp24916_c0_seq1 | No hitsfound | A9YME0*Microctonushyperodae* | 15 | 772 |  |
| comp22969_c0_seq1 | Heat shock 70 kDa protein cogn 5  P29845 *Drosophila* | K7JAU7*Nasoniavitripennis* | 13 | 682 |  |
| comp24894_c0_seq1 | No hitsfound | E1ZZB9*Camponotusfloridanus* | 15 | 650 |  |
| comp22390_c0_seq1 | No hitsfound | V9I8K5*Apiscerana* | 10 | 531 |  |
| **Band 11** |  |  |  |  |  |
| comp24797_c0_seq1 | Venom carboxylesterase-6  B2D0J5 *Apismellifera* | F4WK54*Acromyrmexechinatior* | 17 | 944 |  |
| comp22102_c0_seq1 | No hitsfound | A9YME0*Microctonushyperodae* | 19 | 878 |  |
| comp23136_c0_seq1 | Aconitatehydratase  Q99KI0 *Musmusculus* | E2BSG2*Harpegnathos saltator* | 9 | 493 |  |
| comp22918_c0_seq1 | No hitsfound | B4JLX4*Drosophila grimshawi* | 9 | 442 |  |
| comp21228_c0_seq1 | No hitsfound | W5JWV3*Anopheles darlingi* | 11 | 416 |  |
| comp24842_c0_seq1 | No hitsfound | E2B3J2*Harpegnathossaltator* | 7 | 404 |  |
| comp24962_c0_seq1 | Protein 5NUC  Q9XZ43 *Lutzomyialongipalpis* | F4WSK5*Acromyrmexechinatior* | 9 | 369 |  |
| **Band 12** |  |  |  |  |  |
| comp23766_c0_seq3 | Glucose-6-phosphate isomerase  P52031 *Drosophila yakuba* | K7J470*Nasoniavitripennis* | 19 | 1030 |  |
| comp22460_c0_seq1 | Pyruvatekinase  O62619 *Drosophila melanogaster* | E2BFQ8*Harpegnathos saltator* | 20 | 975 |  |
| comp18004_c0_seq1 | Cytosol aminopeptidase  P00727 *Bostaurus* | V9IKK5*Apiscerana* | 11 | 661 |  |
| comp24274_c0_seq2 | 60 kDa heat shock protein  O02649 *Drosophila melanogaster* | V9II55*Apiscerana* | 10 | 633 |  |
| comp24797_c0_seq1 | Venomcarboxylesterase  B2D0J5 *Apis mellifera* | F4WK54*Acromyrmexechinatior* | 7 | 413 |  |
| **Band 13** |  |  |  |  |  |
| comp17508_c0_seq1 | Tubulin beta-1 chain  O17449 *Manduca sexta* | V9IK78*Apiscerana* | 17 | 945 |  |
| comp24957_c0_seq1 | ATP synthase subunit beta  P56480 *Musmusculus* | V9IJZ2*Apiscerana* | 17 | 935 |  |
| comp24670_c0_seq2 | Alanine aminotransferase 2-like  Q6NYL5 *Daniorerio* | E2B482*Harpegnathos saltator* | 17 | 846 |  |
| comp25089_c0_seq1 | V-type proton ATPase subunit BP31401 *Manducasexta* | E2BNJ3*Harpegnathos saltator* | 13 | 750 |  |
| comp23641_c0_seq2 | Tubulin alpha-1 chain P06603 *Drosophila melanogaster* | K7IY21*Nasoniavitripennis* | 11 | 690 |  |
| comp24952_c0_seq1 | ATP synthase subunit alpha  P35381 *Drosophila melanogaster* | E2BDN0*Harpegnathos saltator* | 12 | 677 |  |
| comp24941_c0_seq1 | Platelet glycoprotein V  O08742 *Musmusculus* | V5GPH6*Anoplophoraglabripennis* | 14 | 675 |  |
| comp21345_c1_seq3 | Proteindisulfide-isomerase  P54399 *Drosophila melanogaster* | E2BG03*Harpegnathos saltator* | 13 | 653 |  |
| comp23157_c0_seq1 | Dihydrolipoyldehydrogenase  P09623 *Sus scrofa* | K7J6V1*Nasoniavitripennis* | 9 | 453 |  |
| comp22812_c0_seq1 | Troponin T  P19351 *Drosophilamelanogaster* | Q3B715*Apis mellifera* | 7 | 391 |  |
| **Band 14** |  |  |  |  |  |
| comp24743_c3_seq1 | Enolase  P15007 *Drosophila melanogaster* | E2A4J2*Camponotusfloridanus* | 13 | 727 |  |
| comp21531_c0_seq1 | Probable citrate synthase 2  Q16P20 *Aedesaegypti* | E2BRV0*Harpegnathos saltator* | 12 | 606 |  |
| comp12881_c0_seq1 | No hitsfound | K7IS29*Nasoniavitripennis* | 10 | 528 |  |
| comp18061_c0_seq1 | Phosphoglycerate kinase  Q01604 *Drosophila melanogaster* | K7IM64*Nasoniavitripennis* | 12 | 586 |  |
| comp22672_c0_seq1 | Leukocyteelastaseinhibitor  Q1JPB0 *Bostaurus* | V9IJT7*Apiscerana* | 7 | 438 |  |
| comp22287_c0_seq1 | Fumaratehydratase  Q60HF9 *Macaca fascicularis* | E2BRN4*Harpegnathos saltator* | 7 | 368 |  |
| comp24914_c0_seq1 | Elongationfactor 1-gamma  P12261 *Artemia salina* | V9II07*Apiscerana* | 7 | 375 |  |
| **Band 15** |  |  |  |  |  |
| comp12889_c0_seq1 | Actin-5C  P84185 *Anopheles gambiae* | S5MIF5*Bombyx mori* | 4 | 385 |  |
| **Band 16** |  |  |  |  |  |
| comp17972_c0_seq1 | Fructose-bisphosphate aldolase  P07764 *Drosophila melanogaster* | K7J4J5*Nasoniavitripennis* | 19 | 1130 |  |
| comp18379_c0_seq1 | Arginine kinase  O61367 *Apis mellifera* | K7IUI5*Nasoniavitripennis* | 17 | 639 |  |
| comp22420_c0_seq1 | Lipase  O46108 *Drosophilamelanogaster* | K7ITV1*Nasoniavitripennis* | 10 | 329 |  |
| comp17775_c0_seq1 | Isocitratedehydrogenase sub beta  Q28479 *Macaca fascicularis* | E2BB73*Harpegnathossaltator* | 8 | 230 |  |
| **Band 17** |  |  |  |  |  |
| comp18379_c0_seq2 | Arginine kinase  O61367 *Apis mellifera* | V9IDW3*Apiscerana* | 20 | 1052 |  |
| comp24345_c0_seq2 | Probableisocitrate DH sub alpha  Q9VWH4 *Drosophilamelanogaster* | K7JCI5*Nasoniavitripennis* | 10 | 462 |  |
| comp17972_c0_seq2 | Fructose-bisphosphate aldolase  P07764 *Drosophila melanogaster* | E2BI91*Harpegnathossaltator* | 9 | 409 |  |
| **Band 18** |  |  |  |  |  |
| comp24967_c0_seq1 | Glyceraldehyde-3-phosphate DH  Q4U3L0 *Glossinamorsitansmorsitans* | Q4PP91*Lysiphlebustestaceipes* | 14 | 725 |  |
| comp25569_c0_seq1 | Tropomyosin  Q1HPU0 *Bombyx mori* | E2C127*Harpegnathossaltator* | 11 | 670 |  |
| comp24996_c0_seq1 | Aldose reductase  P16116 *Bostaurus* | F4WLB5*Acromyrmexechinatior* | 7 | 422 |  |
| **Band 19** |  |  |  |  |  |
| comp25057_c0_seq1 | Malate dehydrogenase  Q5NVR2 *Pongo abelii* | K7J833*Nasoniavitripennis* | 16 | 905 |  |
| comp21083_c0_seq1 | Regucalcin  Q6TLF6 *Danio rerio* | E2B6Z8*Harpegnathos saltator* | 8 | 414 |  |
| comp24696_c0_seq13 | Tropomyosin  Q9NG56 *Blattella germanica* | I4DLW2*Papiliopolytes* | 8 | 390 |  |
| comp22421_c0_seq1 | 1,5-anhydro-D-fructose reductase  Q5U1Y4 *Rattusnorvegicus* | F4WLB4*Acromyrmexechinatior* | 6 | 340 |  |
| **Band 20** |  |  |  |  |  |
| comp16818_c0_seq2 | Chymotrypsin  Q17025 *Anophelesgambiae* | X5MPI4*Locusta migratoria* | 8 | 500 |  |
| comp18379_c0_seq2 | Arginine kinase  O61367 *Apis mellifera* | G3FP78*Bombushypocrita* | 9 | 479 |  |
| comp22432_c0_seq1 | Electron transfer flavoprotein sub beta  P38117 *Homo sapiens* | E2AIR9*Camponotusfloridanus* | 7 | 295 |  |
| **Band 21** |  |  |  |  |  |
| comp20958_c0_seq1 | Granzyme  P08882 *Musmusculus* | X5MPI4*Locusta migratoria* | 13 | 574 |  |
| comp20175_c0_seq1 | 14-3-3 protein zeta  Q2F637 *Bombyx mori* | G9I542*Apisflorea* | 8 | 440 |  |
| comp21177_c0_seq1 | Probable enoyl-CoA hydratase  P34559 *Caenorhabditiselegans* | T1PAX8*Musca domestica* | 4 | 245 |  |
| **Band 22** |  |  |  |  |  |
| comp18379_c0_seq1 | Triosephosphateisomerase  P82204 *Bombyx mori* | K7J7M3*Nasoniavitripennis* | 10 | 496 |  |
| comp18288_c0_seq1 | Phosphoglyceratemutase  O70250 *Musmusculus* | E2BNH7*Harpegnathos saltator* | 11 | 466 |  |
| comp24954_c0_seq1 | Peroxiredoxin-6  Q5ZJF4 *Gallus gallus* | V9IET2*Apiscerana* | 9 | 390 |  |
| comp24987_c0_seq1 | 3-oxoacyl-[ACP] reductaseFabG  Q9X248 *Thermotogamaritima* | V9IJ47*Apiscerana* | 5 | 346 |  |
| **Band 23** |  |  |  |  |  |
| comp24806_c0_seq1 | Venomallergen  P86870 *Vespa magnifica* | P86870*Vespa magnifica* | 11 | 585 |  |
| comp24871_c0_seq1 | Chymotrypsin  Q27289 *Anopheles gambiae* | K7J826*Nasoniavitripennis* | 7 | 390 |  |
| comp22915_c0_seq2 | Heat shock protein beta-1  O13224 *Poeciliopsislucida* | V9IAP4*Apiscerana* | 5 | 367 |  |
| **Band 26** |  |  |  |  |  |
| comp25028_c0_seq1 | Cofilin/actin-depolymer factor homolog  P45594 *Drosophila* | K7IPR6*Nasoniavitripennis* | 10 | 519 |  |
| comp22364_c1_seq2 | Phospholipase A2 (PA3A/PA3B/PA5)  P16354 *Helodermasuspectum* | C4WRN1*Acyrthosiphonpisum* | 10 | 390 |  |
| comp16748_c0_seq1 | Superoxidedismutase [Cu-Zn]  P28755*Ceratitis capitata* | K7IYG5*Nasoniavitripennis* | 7 | 405 |  |
| comp24955_c0_seq1 | Peptidyl-prolyl cis-trans isomerase  P54985*Blattella germanica* | K7J0P3*Nasoniavitripennis* | 8 | 386 |  |
|  |  |  |  |  |  |
| **Band 27** |  |  |  |  |  |
| comp24796_c0_seq1 | No hitsfound | T1H8Z3*Rhodniusprolixus* | 11 | 648 |  |
| comp18097_c0_seq1 | No hits found | B4HT21*Drosophila* | 5 | 309 |  |
| comp17530_c0_seq1 | No hits found | B5DI21*Drosophilapseudoobscura* | 7 | 304 |  |
| comp24825_c0_seq1 | No hits found | W8C879*Ceratitis capitata* | 7 | 281 |  |
| comp16285_c0_seq1 | Cytochrome c-  2P84029*Drosophila* | K7IXL7*Nasoniavitripennis* | 5 | 266 |  |
