# Supplementary material for: Venomics of the ectoparasitoid wasp Bracon nigricans
Source: BMC Genomics. 2020 Jan 10;21:34. doi: 10.1186/s12864-019-6396-4 (PMC6954513; doi:10.1186/s12864-019-6396-4)
Supplement: Supplementary file 4 — Additional file 4: Table S3. Homologs of B. nigricans venom proteins occurring in other parasitoid species [file 12864_2019_6396_MOESM4_ESM.docx]

**Table S3. Homologs of *Bracon nigricans* venom proteins occurring in other parasitoid species** (V: venom; VG: venom glands; T: teratocytes; LS: larval saliva).

| Species | Functional annotation reported | Localization | References |
| --- | --- | --- | --- |
| *Anisopteromalus calandrae* (Ac) | Odorant binding protein, Carboxylesterase | VG | [1] |
| *Aphidius ervi* (Ae) | Protein disulphide isomerase, Leucine rich repeat | VG | [2] |
| *Bracon hebetor* (Bh) | Phospholipase A2, Chymotrypsin, Prokaryotic membrane lipoprotein lipid attachment site profile | V | [3] |
| *Chelonus inanitus* (Ci) | Odorant binding protein, Venom allergen 5, Lipase | V/VG | [4] |
| *Cotesia chilonis* (Cc) | Protein disulphide isomerase | V/VG | [5] |
| *Cotesia rubecula* (Cr) | Chymotrypsin | V/VG | [6] |
| *Dinocampus coccinellae* (Dc) | Carboxylesterase | T | [7] |
| *Diversinervus elegans* (De) | Alpha mannosidase, Protein disulphide isomerase | V/VG | [8] |
| *Eupelmus orientalis* (Eo) | Phospholipase A2 | V | [9] |
| *Euplectrus separatae* (Es) | Chymotrypsin | LS | [10] |
| *Hyposoter didymator* (Hd) | Carboxylesterase, Venom allergen 5 | V | [11] |
| *Leptopilina boulardi* (Lb) | Lipase, Aminopeptidase N-type | V/VG | [12] |
| *Leptopilina heterotoma* (Lh) | Odorant Binding Protein | VG | [13] |
| *Microctonus aethiopoides* (Ma) | Lipase | V/VG | [14] |
| *Microctonus hyperodae* (Mh) | DUF4803, Venom allergen 5 | V/VG | [14] |
| *Nasonia vitripennis* (Nv) | Carboxylesterase, Chymotrypsin, Venom allergen 5, Odorant binding protein | V | [15] |
| *Ooencyrtus telenomicida* (Ot) | Lipase | VG | [16] |
| *Pimpla hypochondriaca* (Ph) | Lipase, Aminopeptidase, Serine protease | V | [17, 18] |
| *Pteromalus puparum* (Pp) | Chymotrypsin, Lipase, Odorant binding protein | V/VG | [19] |
| *Psyttalia concolor* (Pc) | Phospholipase A2, DUF4803, Protein disulphide isomerase, Leucine rich repeat | V/VG | [20] |
| *Psyttalia lounsburyi* (Pl) | DUF4803, Protein disulphide isomerase, Leucine rich repeat | V/VG | [20] |
| *Tetrastichus brontispae* (Tb) | Protein disulphide isomerase, Venom allergen 5, Odorant binding protein | V | [21] |
| *Toxoneuron nigriceps* (Tn) | Phospholipase A2 | V/VG | [22] |

1. Perkin LC, Friesen KS, Flinn PW, Oppert B. Venom gland components of the ectoparasitoid wasp, Anisopteromalus calandrae. OPEN ACCESS. 2015;6:19.

2. Colinet D, Anselme C, Deleury E, Mancini D, Poulain J, Azéma-Dossat C, et al. Identification of the main venom protein components of *Aphidius ervi*, a parasitoid wasp of the aphid model *Acyrthosiphon pisum*. BMC Genomics. 2014;15:342.

3. Windass JD, Duncan RE, Baule VJ, Christian PD. Toxins from the wasp *Bracon hebetor*. 1996;:WO1996016171A1. https://patents.google.com/patent/WO1996016171A1/en. Accessed 9 Apr 2019.

4. Vincent B, Kaeslin M, Roth T, Heller M, Poulain J, Cousserans F, et al. The venom composition of the parasitic wasp *Chelonus inanitus* resolved by combined expressed sequence tags analysis and proteomic approach. BMC Genomics. 2010;11:693.

5. Teng Z-W, Xiong S-J, Xu G, Gan S-Y, Chen X, Stanley D, et al. Protein Discovery: Combined Transcriptomic and Proteomic Analyses of Venom from the Endoparasitoid Cotesia chilonis (Hymenoptera: Braconidae). Toxins. 2017;9. doi:10.3390/toxins9040135.

6. Asgari S, Zhang G, Zareie R, Schmidt O. A serine proteinase homolog venom protein from an endoparasitoid wasp inhibits melanization of the host hemolymph. Insect Biochem Mol Biol. 2003;33:1017–24.

7. Gopalapillai R, Kadono-Okuda K, Okuda T. Molecular cloning and analysis of a novel teratocyte-specific carboxylesterase from the parasitic wasp, *Dinocampus coccinellae*. Insect Biochem Mol Biol. 2005;35:1171–80.

8. Liu N-Y, Wang J-Q, Zhang Z-B, Huang J-M, Zhu J-Y. Unraveling the venom components of an encyrtid endoparasitoid wasp *Diversinervus elegans*. Toxicon. 2017;136:15–26.

9. Doury G, Bigot Y, Periquet G. Physiological and biochemical analysis of factors in the female venom gland and larval salivary secretions of the ectoparasitoid wasp *Eupelmus orientalis*. J Insect Physiol. 1997;43:69–81.

10. Nakamatsu Y, Tanaka T. The function of a trypsin-like enzyme in the saliva of *Euplectrus separatae* larvae. J Insect Physiol. 2004;50:847–54.

11. Dorémus T, Urbach S, Jouan V, Cousserans F, Ravallec M, Demettre E, et al. Venom gland extract is not required for successful parasitism in the polydnavirus-associated endoparasitoid Hyposoter didymator (Hym. Ichneumonidae) despite the presence of numerous novel and conserved venom proteins. Insect Biochem Mol Biol. 2013;43:292–307.

12. Colinet D, Deleury E, Anselme C, Cazes D, Poulain J, Azema-Dossat C, et al. Extensive inter- and intraspecific venom variation in closely related parasites targeting the same host: The case of Leptopilina parasitoids of Drosophila. Insect Biochem Mol Biol. 2013;43:601–11.

13. Heavner ME, Gueguen G, Rajwani R, Pagan PE, Small C, Govind S. Partial venom gland transcriptome of a *Drosophila* parasitoid wasp, *Leptopilina heterotoma*, reveals novel and shared bioactive profiles with stinging Hymenoptera. Gene. 2013;526:195–204.

14. Crawford AM, Brauning R, Smolenski G, Ferguson C, Barton D, Wheeler TT, et al. The constituents of *Microctonus* sp. parasitoid venoms. Insect Mol Biol. 2008;17:313–24.

15. de Graaf DC, Aerts M, Brunain M, Desjardins CA, Jacobs FJ, Werren JH, et al. Insights into the venom composition of the ectoparasitoid wasp *Nasonia vitripennis* from bioinformatic and proteomic studies. Insect Mol Biol. 2010;19:11–26.

16. Cusumano A, Duvic B, Jouan V, Ravallec M, Legeai F, Peri E, et al. First extensive characterization of the venom gland from an egg parasitoid: structure, transcriptome and functional role. J Insect Physiol. 2018;107:68–80.

17. Dani MP, Edwards JP, Richards EH. Hydrolase activity in the venom of the pupal endoparasitic wasp, Pimpla hypochondriaca. Comp Biochem Physiol B Biochem Mol Biol. 2005;141:373–81.

18. Parkinson N, Richards EH, Conyers C, Smith I, Edwards JP. Analysis of venom constituents from the parasitoid wasp Pimpla hypochondriaca and cloning of a cDNA encoding a venom protein. Insect Biochem Mol Biol. 2002;32:729–35.

19. Yan Z, Fang Q, Wang L, Liu J, Zhu Y, Wang F, et al. Insights into the venom composition and evolution of an endoparasitoid wasp by combining proteomic and transcriptomic analyses. Sci Rep. 2016;6:19604.

20. Mathé-Hubert H, Colinet D, Deleury E, Belghazi M, Ravallec M, Poulain J, et al. Comparative venomics of *Psyttalia lounsburyi* and *P. concolor*, two olive fruit fly parasitoids: a hypothetical role for a GH1 β-glucosidase. Sci Rep. 2016;6:35873.

21. Liu N-Y, Xu Z-W, Yan W, Ren X-M, Zhang Z-Q, Zhu J-Y. Venomics reveals novel ion transport peptide-likes (ITPLs) from the parasitoid wasp *Tetrastichus brontispae*. Toxicon Off J Int Soc Toxinology. 2018;141:88–93.

22. Laurino S, Grossi G, Pucci P, Flagiello A, Bufo SA, Bianco G, et al. Identification of major *Toxoneuron nigriceps* venom proteins using an integrated transcriptomic/proteomic approach. Insect Biochem Mol Biol. 2016;76:49–61.
