# Supplementary material for: Venomics of the ectoparasitoid wasp Bracon nigricans
Source: BMC Genomics. 2020 Jan 10;21:34. doi: 10.1186/s12864-019-6396-4 (PMC6954513; doi:10.1186/s12864-019-6396-4)
Supplement: Supplementary file 5 — Additional file 5: Table S4. One-way ANOVA of ΔCt values recorded in venom glands, females devoid of venom glands and males [file 12864_2019_6396_MOESM5_ESM.docx]

**Table S4. One-way ANOVA of ΔCt values recorded in: venom glands, females devoid of venom glands and males.**

| Transcript | F | Significance |
| --- | --- | --- |
| Protein disulphide isomerase | F_2,8_=118.255 | *P* <0.001 |
| Lysosomal alpha-mannosidase | F_2,5_=23.58 | *P*<0.005 |
| Phospholipase A2 | F_2,6_=137.03 | *P*<0.001 |
| Leucyl-cystinil aminopeptidase | F_2,6_=167.765 | *P*<0.001 |
| Lipase | F_2,6_=62.89 | *P*<0.001 |
| Odorant binding protein | F_2,7_=255.488 | *P*<0.001 |
| Trypsin-like serine protease | F_2,6_=94.73 | *P*<0.001 |
| Carboxylesterase | F_2,5_=1122.740 | *P*<0.001 |
