# Supplementary material for: Venomics of the ectoparasitoid wasp Bracon nigricans
Source: BMC Genomics. 2020 Jan 10;21:34. doi: 10.1186/s12864-019-6396-4 (PMC6954513; doi:10.1186/s12864-019-6396-4)

Fig. S2. Amino acid sequence alignment of representative phospholipases A2 from venomous and non-venomous species. *Bn*PLA2 was aligned with putative homologues from the following species: *Bracon hebetor* (CAB42203.1), *Ampulex compressa* (ARK19802.1[a], ARK19891.1[b], ARK19877.1[c]), *Eumenes pomiformis* (ACZ37401.1), *Orancistrocerus drewseni* (ACD61710.3), *Apis mellifera* (AFI40558.1), *Drosophila melanogaster* (NP_001137612.1), *Heloderma suspectum* (P16354.3), *Bos taurus* (Q1JPB9.1), *Homo sapiens* (Q9NZ20.2).The active sites of PLA2s are indicated by a red triangle, while calcium binding sites are indicated by a blue dot.

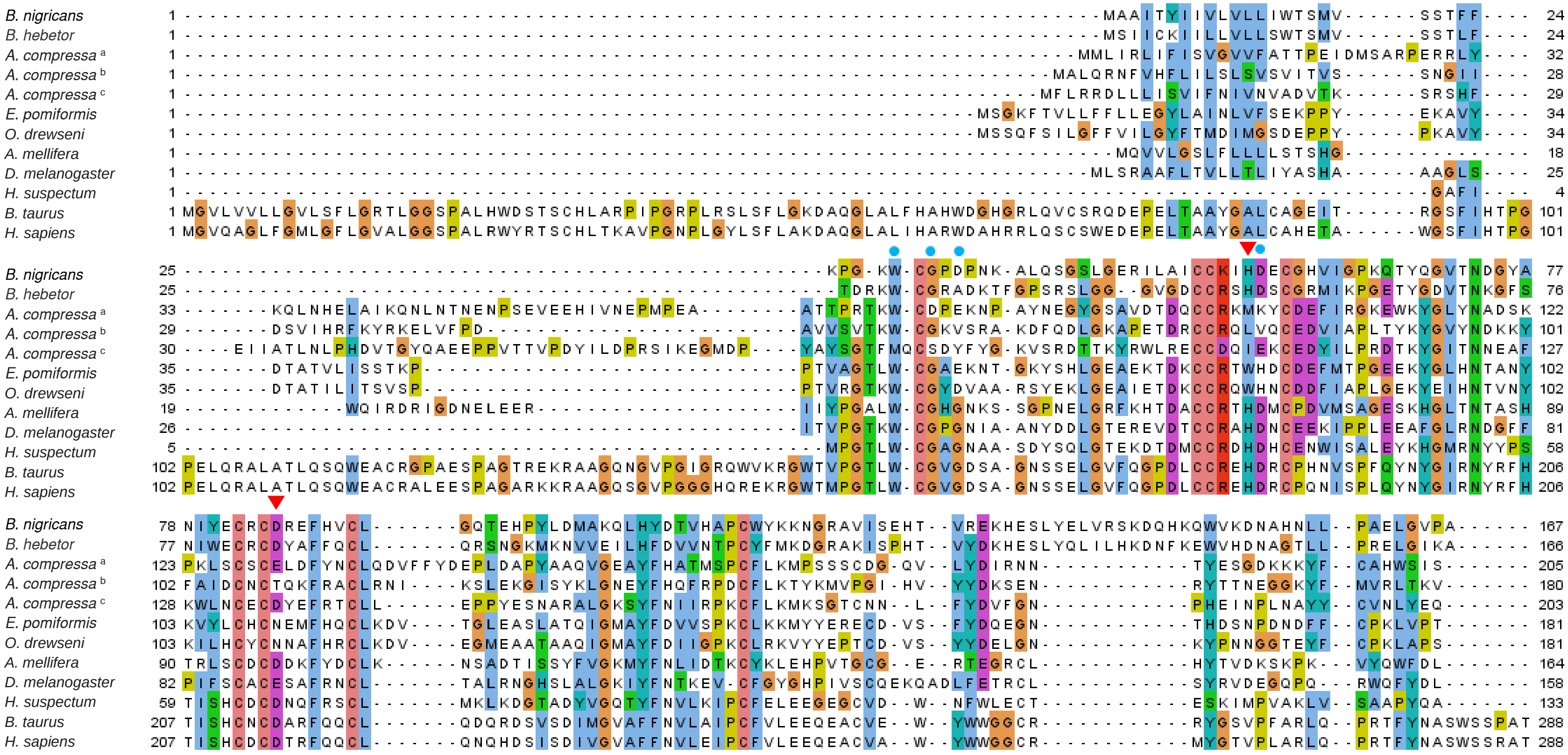

Fig. S3. Amino acid sequence alignment of representative lipases from venomous and non-venomous species. *Bn*LIP was aligned with putative homologues from the following species: *Nasonia vitripennis* (XP_008216585.2), *Fopius arisanus* (XP_011301786.1), *Drosophila melanogaster* (O46107.2), *Crotalus adamanteus* (J3SDX8), *Homo sapiens* (Q5VXJ0.2). The active sites of lipases are indicated by a red triangle.

```
B. nigricans      1 --------------------------------------------------------------------MNFAFGFLLTFWYLN  15
N. vitripennis    1 ----------MHSLPASPSDSIGPPQTQSQGMYPRNSIMNGVRLHFVLCLAALCLVQETSTFILDLSPAKFARERYERSRNKT  73
F. arisanus       1 ----------------------------------------------------------------------MNMFSIVICLTQL  13
D. melanogaster   1 --------------------------------MRCSLRMQLLLLLGLCVFISRIQGQLIGGEEDEEDEEEEEEEEESVEDETP  51
C. adamanteus     1 -----------------------------------------------------------------MWRLIIIAILFQGLVNSA  18
H. sapiens        1 -----------------------------------------------------------------MWQLLAAACWMLLLGSMY  18

B. nigricans     16 AGTFARPSGIDSQPLKFTELIKHFGYTGESHWVTTSDGYILRVDRITSGPSTP-AAEGKPVVFLMHGITGASEHFVFWERSTS  97
N. vitripennis   74 TARTFTPVPNYVQAVDFVSLVNRHGYPGEEHVVMTADGYLLRIHRIPGSPSRP-RAVGKPVIYMQHGLLASSDTWVLMGPQRD 155
F. arisanus      14 FALGVSKPVAFKGHLNFTELVEQYGYTPESHVVTTEDGYNIRLDRISGAPTSP-EAPGKPVVYLLHGLGVASEAWILWTPNNS  95
D. melanogaster  52 EDRLQRKNIKQDSTLSVDKLIAKYGYESEVHHVTTEDGYILTMHRI--------RKQGAPPFLLQHGLVDSSAGFVVMGPNVS 126
C. adamanteus    19 MLERRKRGVDPETAMNISEIILFRGYPSEEYEVVTGDGYILCLNRIPYGKISQKTKEPKPAVFLQHGLLADGSNWVTNLDYNS 101
H. sapiens       19 GYDKKGNNANPEANMNISQIISYWGYPYEEYDVTTKDGYILGIYRIPHGRGCPGRTAPKPAVYLQHGLIASASNWICNLPNNS 101

B. nigricans     98 LAYLLVDSGYDVWLGNNRGNTNSRNHTTLPP-DARPFWNFTLTDLGTKDLPTMIDFVLTKTGQQRLAYVGHSCGTTISYILLS 179
N. vitripennis  156 LAYILADAGYDVWLGNVRGNTYSRAHVSLSPDYDPAFWEFSYHEIALYDVTAVIDYILIKTAQPSLVYIGHSMGTTISYILLS 238
F. arisanus      96 LAFLLADAGYDVWLGNIRGTTHGRSHQSLDPDKDREFWNFDLTTISTMDLPAQVDFILEKTGESKLTIIGHSMGTTLSYIFLS 178
D. melanogaster 127 LAYLLADHNYDVWLGNARGNRYSRNHTTLDP-DESKFWDFSWHEIGMYDLPAMIDHVLKVTGFPKLHYAGHSQGCTSFFVMCS 208
C. adamanteus   102 LGFALADAGFDVWLGNSRGNTWSQKHINYTI-KQKEFWMFSFNEMAMYDIPASVNFVLNKTGQEQLFYVGHSQGTTIGFIAFS 183
H. sapiens      102 LAFLLADSGYDVWLGNSRGNTWSRKHLKLSP-KSPEYWAFSLDEMAKYDLPATINFIIEKTGQKRLYYVGHSQGTTIAFIAFS 183

B. nigricans    180 ELPQYNEKMSVVVSLAPVALW-----NPSSVFFLKMMANAAVK---FFQVTLAADDYSVEIFSSSSLTSSVCNFWNQAICLWL 254
N. vitripennis  239 IKPEYNKKIRLLVSLAPVAFW-HAPPRAFVRFLIDNTEAIKSF---VTNARIYELFPLTAANAQLAKTTCSDGSIFQQLCMNF 317
F. arisanus     179 EKPTYNNKVNLVVSLAPIAYW-HPKTLGIIRIFMNAAFETAKL---LVAGPDINAEVMPHGPHVVKFADDYCNPNISDICLKF 257
D. melanogaster 209 MRPAYNDKVVSMQALAPAVYAKETEDHPYIRAISLYFNSLVGS-------------SIREMFNGEFRFLCRMTEETERLCIEA 278
C. adamanteus   184 VLPELAKKIKMFFGLAPVMTV-KFSSGGLVKLGELPEFLLKEI---F----GTKQIFPQNAVIKWLATHVCGQVLIDELCGNF 258
H. sapiens      184 TNPELAKKIKIFFALAPVVTV-KYTQSPMKKLTTLSRRVVKVL---F----GDKMFHPHTLFDQFIATKVCNRKLFRRICSNF 258

B. nigricans    255 LNTFLGHPSVPFSDDDFKYVAMYVPAGSSLKVFNHFRQNINSASFANYDYGNAAANRKQYGQDKPPLFDLKKITAPVAVFYSY 337
N. vitripennis  318 VYYSSGYNPEQLNASEVSYVLSYFPAGTSAQTLIHFSQNMRTGDFQMYDHGF-IRNLATYKQRQPPMYNLSNIISPVGLFYGK 399
F. arisanus     258 ANIFMQKTTSPLSFEELRHLMEYAPAGSSLRLHHHYLQNINSGNFQKYDYESTAANIEHYGQETPSKYNLSNIDTPVVLIYIK 340
D. melanogaster 279 VFGIVGRNWNEFNRKMFPVILGHYPAGVAAKQVKHFIQIIKSGRFAPYSYSS-NKNMQLYRDHLPPRYNLSLVTVPTFVYYST 360
C. adamanteus   259 FFLLCGFNEKNLNMSRVEIYSTHCPAGTSVQNMLHWSQAVKSGEVRAFDWGSRKENMAHYKQPTPPPYKMERMLVPTALWTGG 341
H. sapiens      259 LFTLSGFDPQNLNMSRLDVYLSHNPAGTSVQNMLHWAQAVNSGQLQAFDWGNSDQNMMHFHQLTPPLYNITKMEVPTAIWNGG 341

B. nigricans    338 NDPVSPKDSIDKLAATLPNFISTNVVNAPTFTHTDYFYHDGMRSLFGDKVVEIINGKSTKIN----------------- 399
N. vitripennis  400 GDALVSPGNPIELSQKLPNVLTIEAVPDEKFSHLDFLWSTDIRKLLNDRIFEFISKTLRQQNSAL-------------- 464
F. arisanus     341 NDFFSPEISTRILEKHLPNIVVSEAVPDKAFVHADFIFHKNVKAVIYDRVIKLIGEILKIERKL--------------- 404
D. melanogaster 361 NDLLCHPKDVESMCDDLGNVTGKYLVPQKEFNHMDFLWAIDVRKMLYRRMLQVLGKVPEGSPEEANRSRREIRGKFIRS 439
C. adamanteus   342 HDWLSDRKDIAILLTLIPNLIYHKEIPE--WEHLDFIWGLDAPQRMFRDMIQMMHKVQYAH------------------ 400
H. sapiens      342 QDIVADPKDVENLLPQIANLIYYKLIPH--YNHVDFYLGEDAPQEIYQDLIILMEEYLQN------------------- 399
```

Fig. S4. Amino acid sequence alignment of representative carboxylesterases from venomous and non-venomous species. Carboxylesterase of *B. nigricans* venom (*Bn*CAR) was aligned with putative homologues from the following species: *Nasonia vitripennis* (XP_001599255.3), *Apis mellifera* (B2D0J5), *Myzus persicae* (P35501.1), *Mus musculus* (Q8BK48.1), *Homo sapiens* (Q8N0W4.1).The active sites of carboxylesterases are indicated by a red triangle, while consensus pattern for Carboxylesterases type-B signature 2 (Prosite accession: PS00941) is indicated by a blue line.

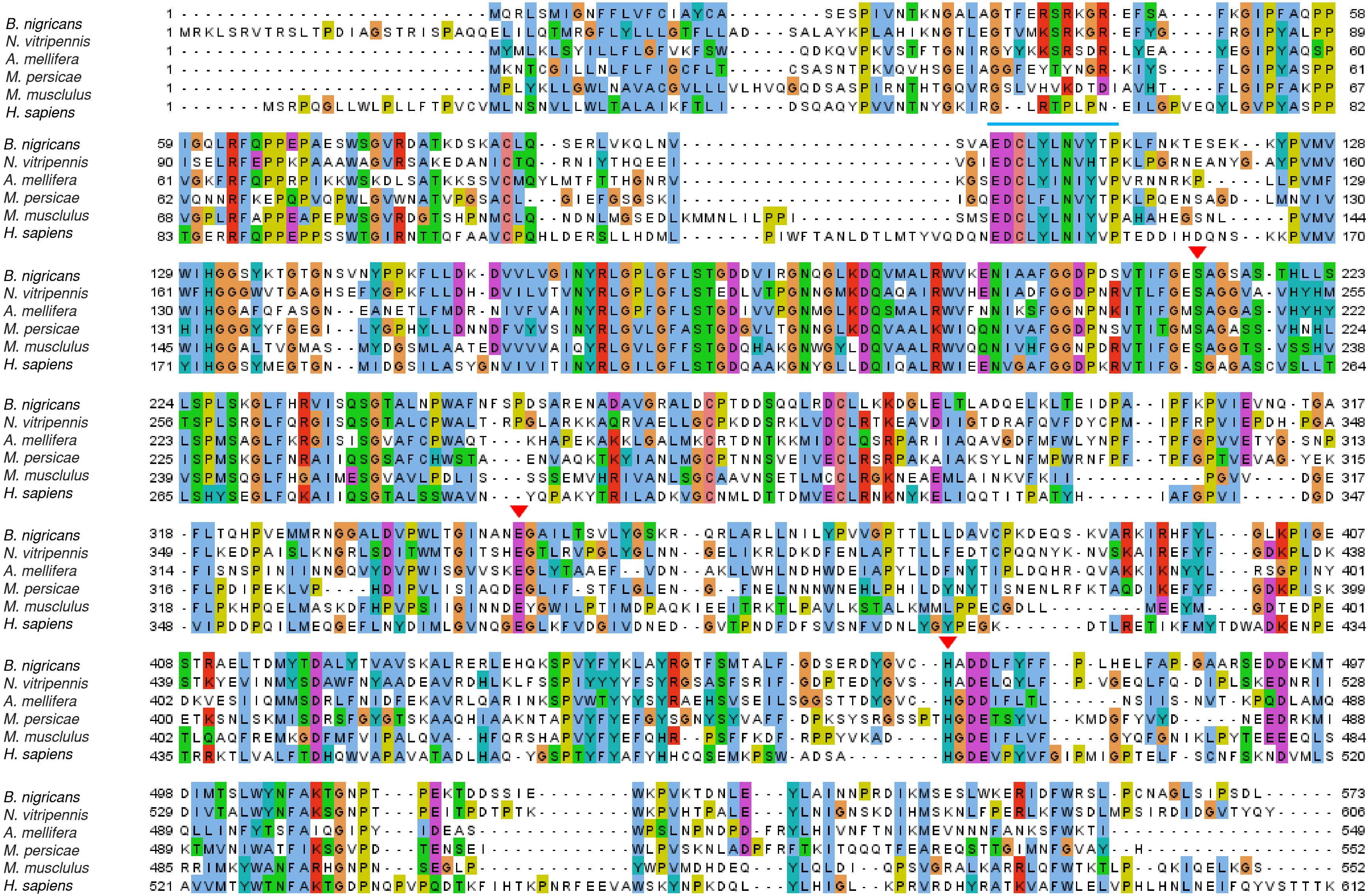

Fig. S5. Amino acid sequence alignment of representative trypsin-like serine proteases from venomous and non-venomous species. Trypsin-like serine protease of *B. nigricans* venom (*Bn*TRY) was aligned with putative homologues from the following species: *Bracon hebetor* (CAB42201.1), *Nasonia vitripennis* (NP_001155042.1), *Apis mellifera* (XP_393127.4), *Anopheles gambiae* (Q17025.3), *Blarina brevicauda* (Q76B45), *Mus* musclulus (P15119), *Rattus norvegicus* (P18291), *Homo sapiens* (Q6GPI1.2).The active sites of serine proteases are indicated by a red triangle.

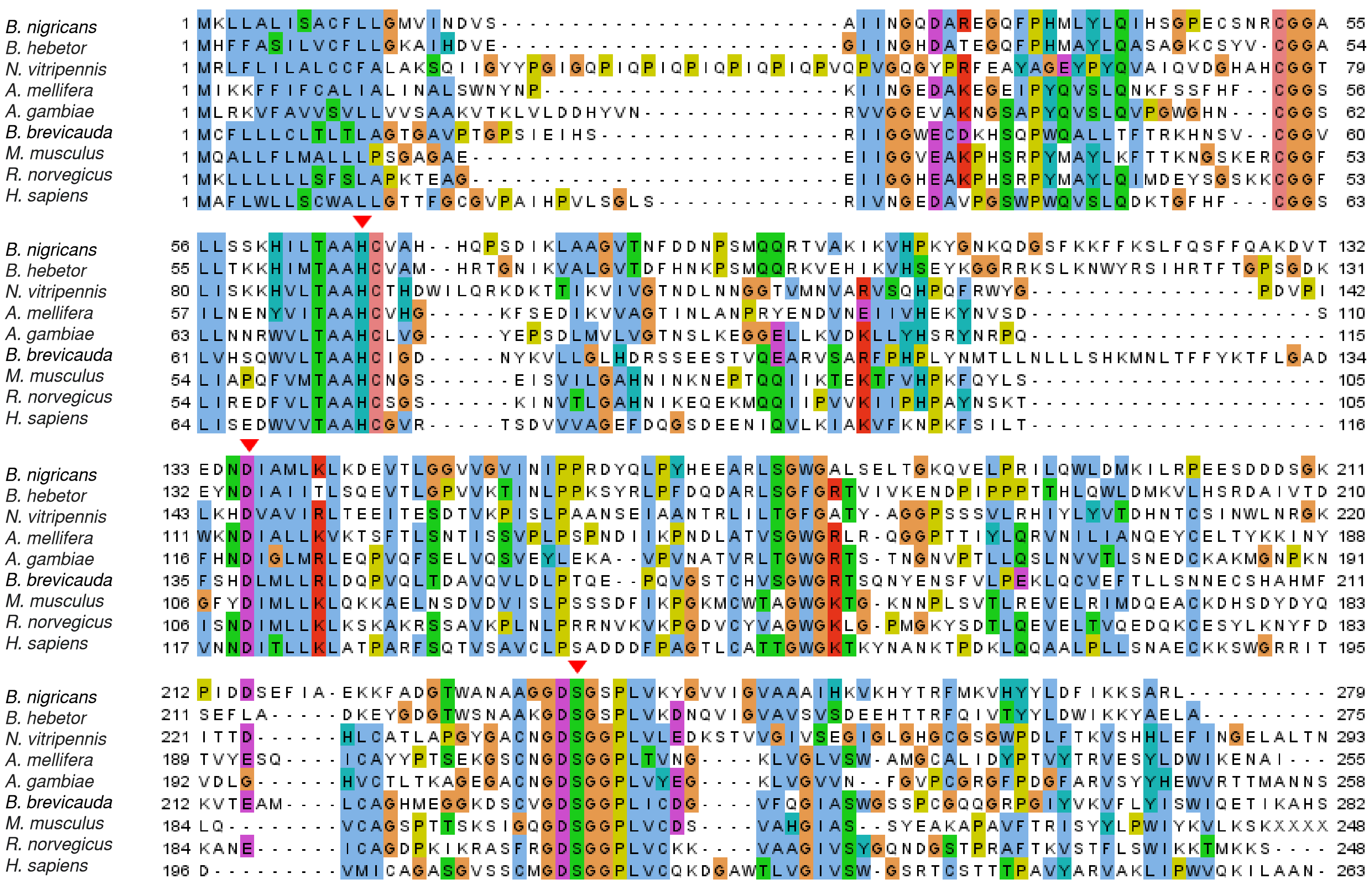

Fig. S6. Amino acid sequence alignment of representative aminopeptidases from venomous and non-venomous species. Leucyl-cystinil aminopeptidase of *B. nigricans* venom (*Bn*LCA) was aligned with putative homologues from the following species: *Microplitis demolitor* (XP_014296891.1), *Manduca sexta* (Q11001.1), *Caenorhabditis elegans* (Q4TT88.1), *Sus scrofa* (Q95334.1), *Homo sapiens* (Q07075.3), *Oryza sativa* (Q6Z6L4.1). The active sites of aminopeptidases are indicated by a red triangle, while zinc binding sites are indicated by a blue dot.

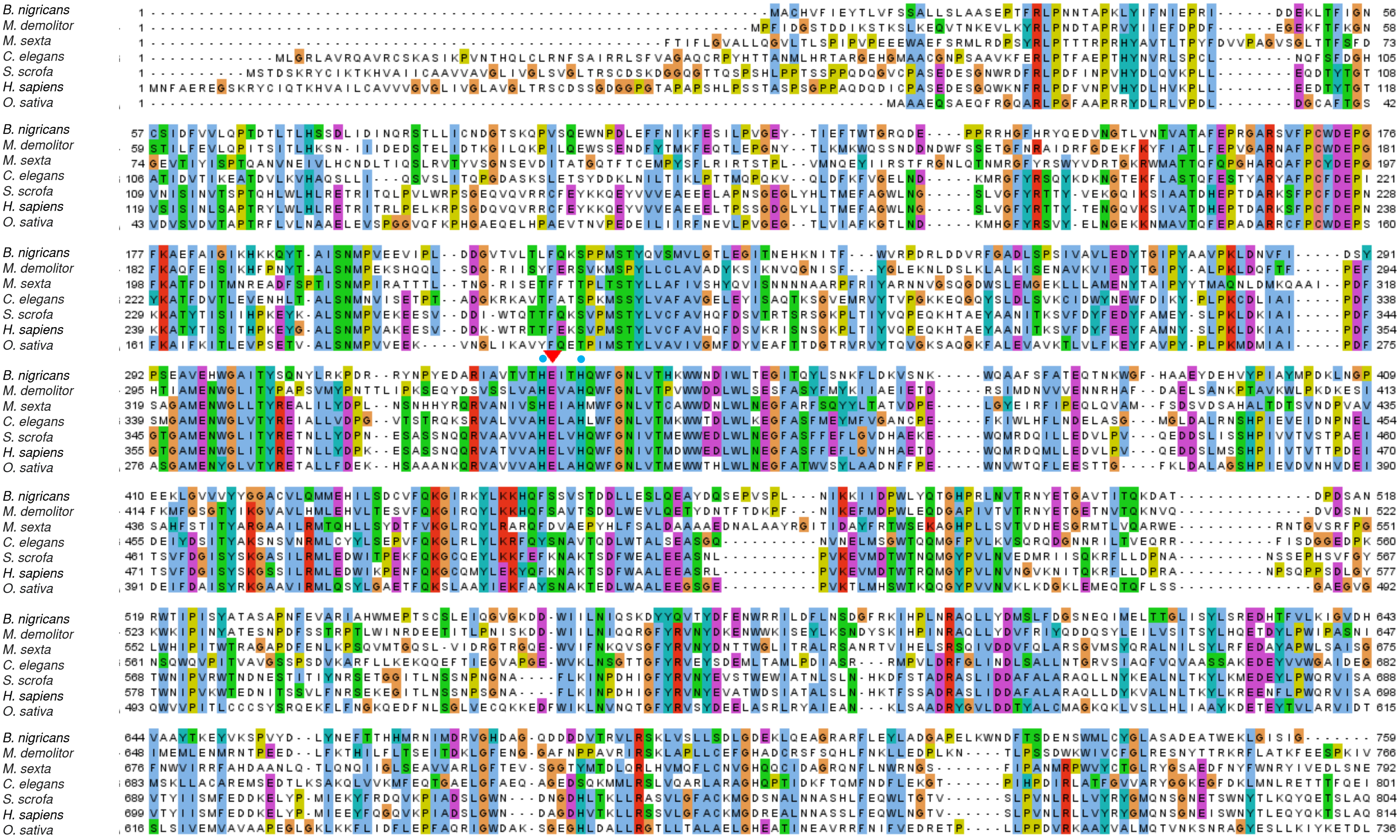

Fig. S7. Amino acid sequence alignment of representative mannosidases from venomous and non-venomous species. Lysosomal alpha-mannosidase of *B. nigricans* venom (*Bn*LAM) was aligned with putative homologues from the following species: *Nasonia vitripennis* (XP_001603702.3), *Drosophila ananassae* (XP_014762200.1), *Bos taurus* (Q29451.4), *Mus musculus* (Q54782.2), *Homo sapiens* (Q9Y2E5.4), *Arabidopsis thaliana* (P94078.1). The active sites of mannosidases are indicated by a red triangle, while zinc binding sites are indicated by a blue dot.

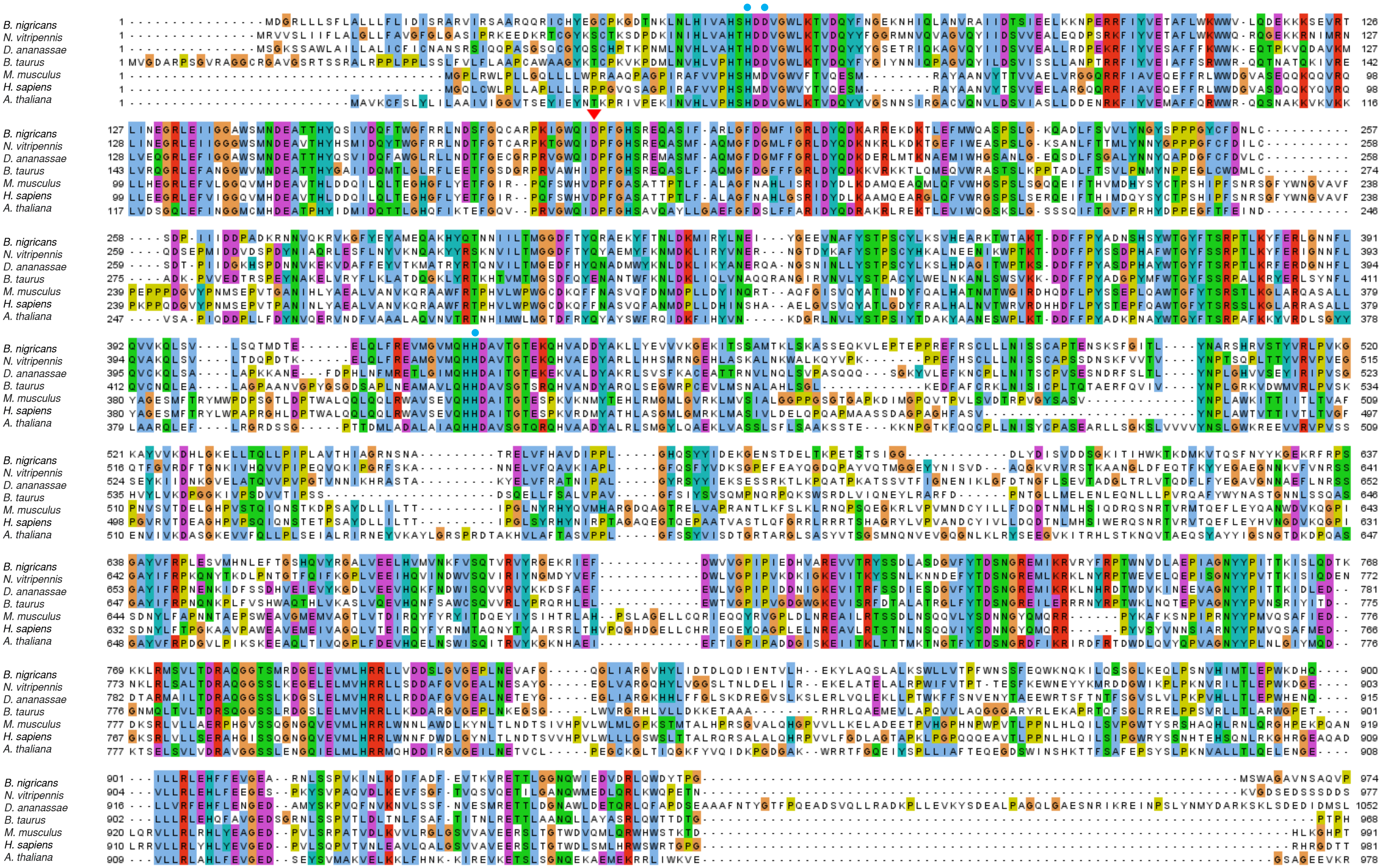

Fig. S8. Amino acid sequence alignment of putative homologous odorant-binding proteins from venomous and non-venomous species. Odorant-binding protein of *B. nigricans* venom (*Bn*OBP) was aligned with putative homologues from the following species: *Bracon hebetor* (AXY94705.1), *Fopius arisanus* (XP_011314550.1), *Nasonia vitripennis* (XP_003425339.1), *Brachigaster minutus* (GBMS01012538.1), *Pteromalus puparum* (GECT01010095.1), *Psyttalia lounsburyi* (GCEQ01015168.1[a], GCEQ01012717.1[b], JZ818750.1[c]), *Ctenocephalides_felis* (XP_026482353.1). Conserved cysteines are indicated by green dots.

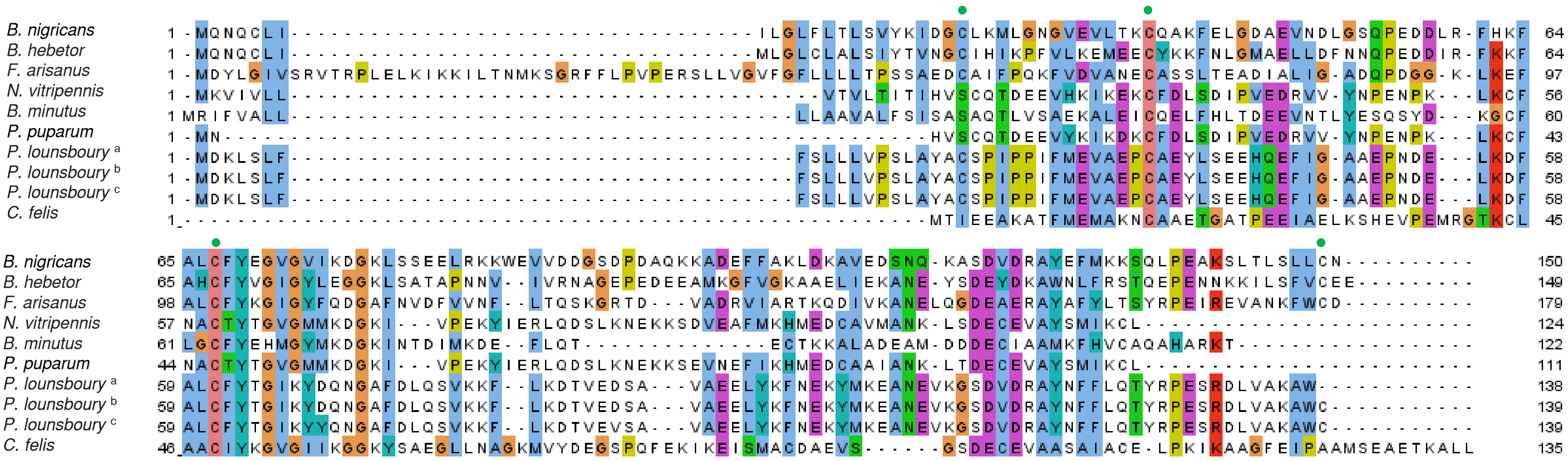

Fig. S9. Amino acid sequence alignment of representative protein disulfide-isomerases from venomous and non-venomous species. Protein disulfide-isomerase of *B. nigricans* venom (*Bn*PDI) was aligned with putative homologues from the following species: *Bracon hebetor* (AXY94695.1), *Microplitis demolitor* (XP_008554924.1), *Apis mellifera* (XP_026298883.1), *Anopheles gambiae* (XP_551775.3), *Drosophila melanogaster* (P54399.1), *Conus varius* (AOZ19959.1), *Mus musculus* (D3Z6P0.1), *Homo sapiens* (Q13087.2). The active sites of protein disulfide-isomerases are indicated by a red triangle, while endoplasmic reticulum targeting sequence (Prosite accession: PS00014) is indicated by a blue line.

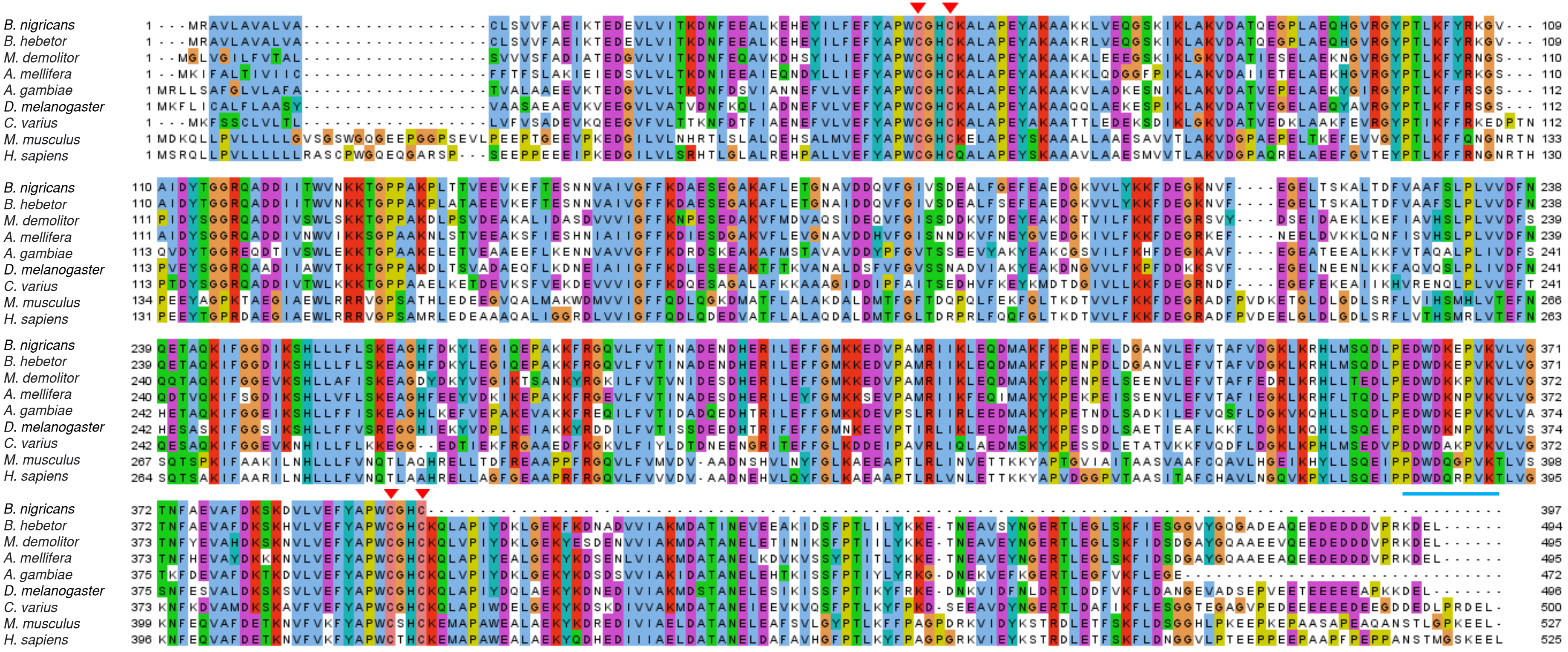

Supplement: Supplementary file 6 — Additional file 6: Figures S2-S9. Multiple sequence alignments of most representative B. nigricans venom proteins [file 12864_2019_6396_MOESM6_ESM.pdf]
