# Supplementary material for: Venomics of the ectoparasitoid wasp Bracon nigricans
Source: BMC Genomics. 2020 Jan 10;21:34. doi: 10.1186/s12864-019-6396-4 (PMC6954513; doi:10.1186/s12864-019-6396-4)

**Fig. S10. Maximum-likelihood tree of phospholipase-A2 amino acid sequences.** Nodes are labeled with bootstrap support. The accession number of each sequence is followed by the taxon name. Phospholipase A2 of *B. nigricans* venom (*Bn*PLA2 ) is indicated by a red arrow. Highlighted branches represent putative homologs of *Bn*PLA2 in Apidae (blue) and hymenopterans with mutated histidine catalytic site (green). Tree is rooted to putative orthologs in Arachnida.

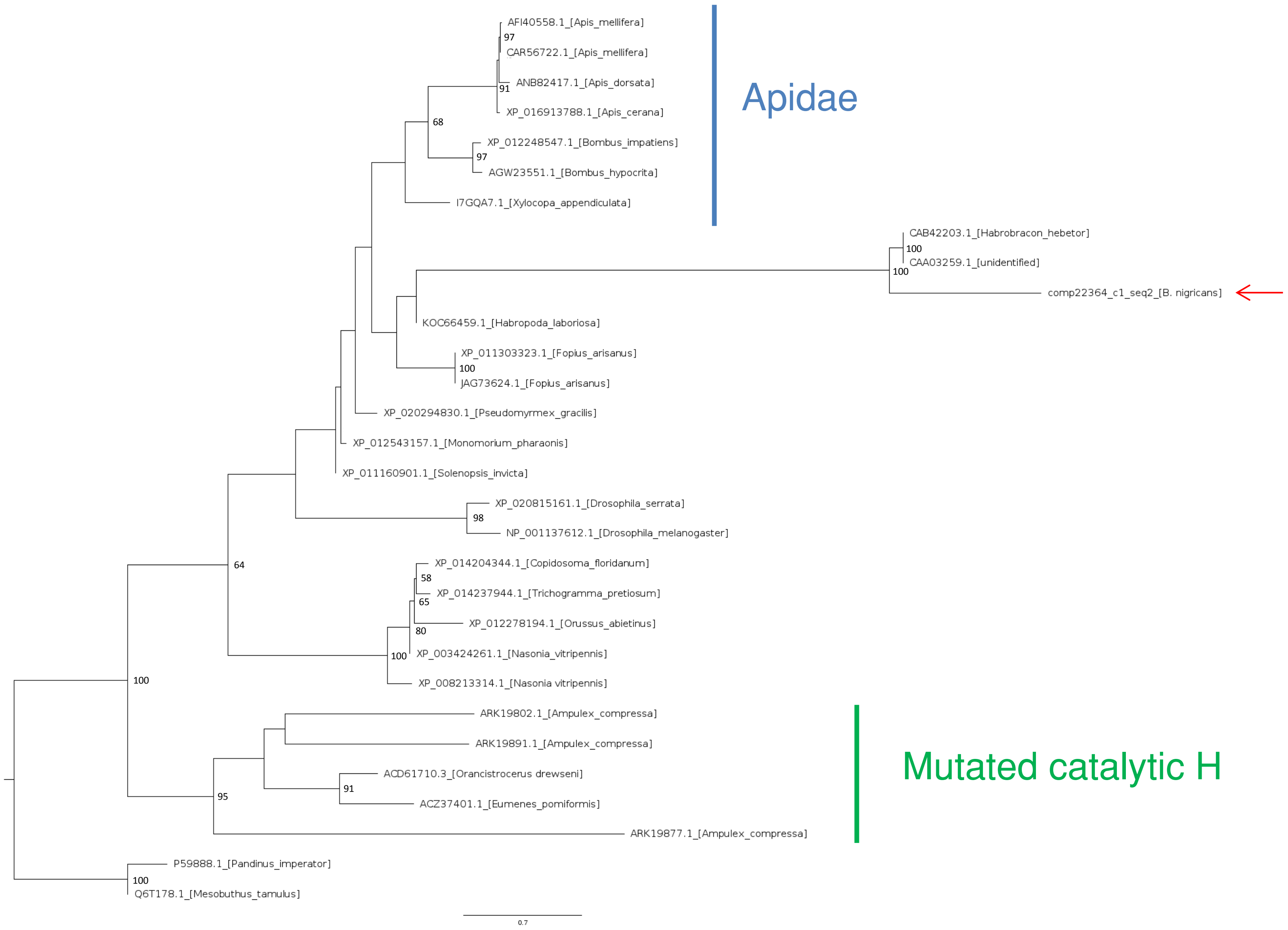

**Fig. S11. Maximum-likelihood tree of lipase amino acid sequences.** Nodes are labeled with bootstrap support. The accession number of each sequence is followed by the taxon name. Lipase of *B. nigricans* venom (*Bn*LIP) is indicated by a red arrow. Highlighted branches represent putative homologs of *Bn*LIP in Apidae (blue) and Formicidae (green). Tree is rooted to putative orthologs in Arachnida.

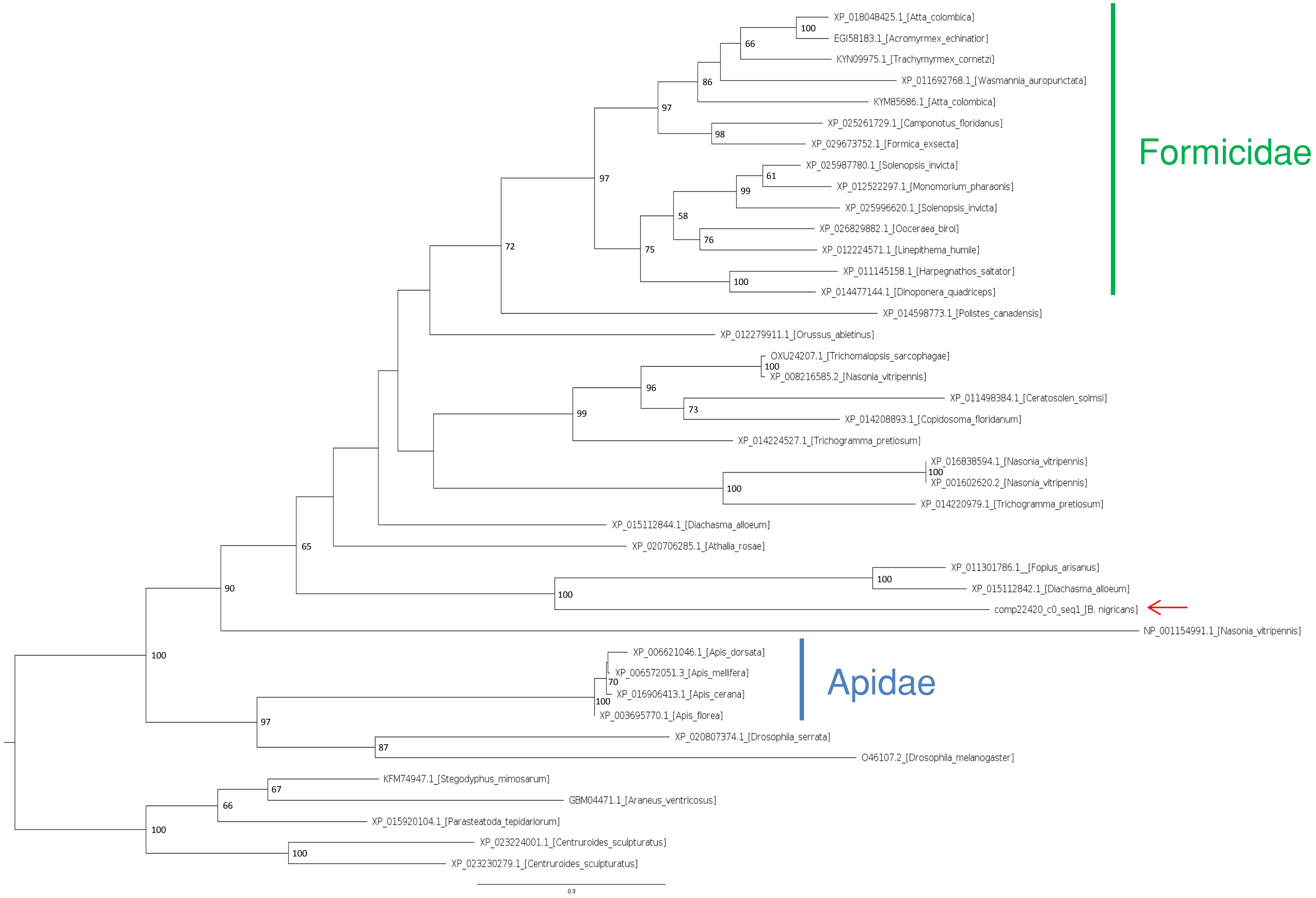

**Fig. S12. Maximum-likelihood tree of carboxylesterase amino acid sequences.** Nodes are labeled with bootstrap support. The accession number of each sequence is followed by the taxon name. Carboxylesterase of *B. nigricans* venom is indicated by a red arrow. Tree is rooted to putative orthologs in Arachnida.

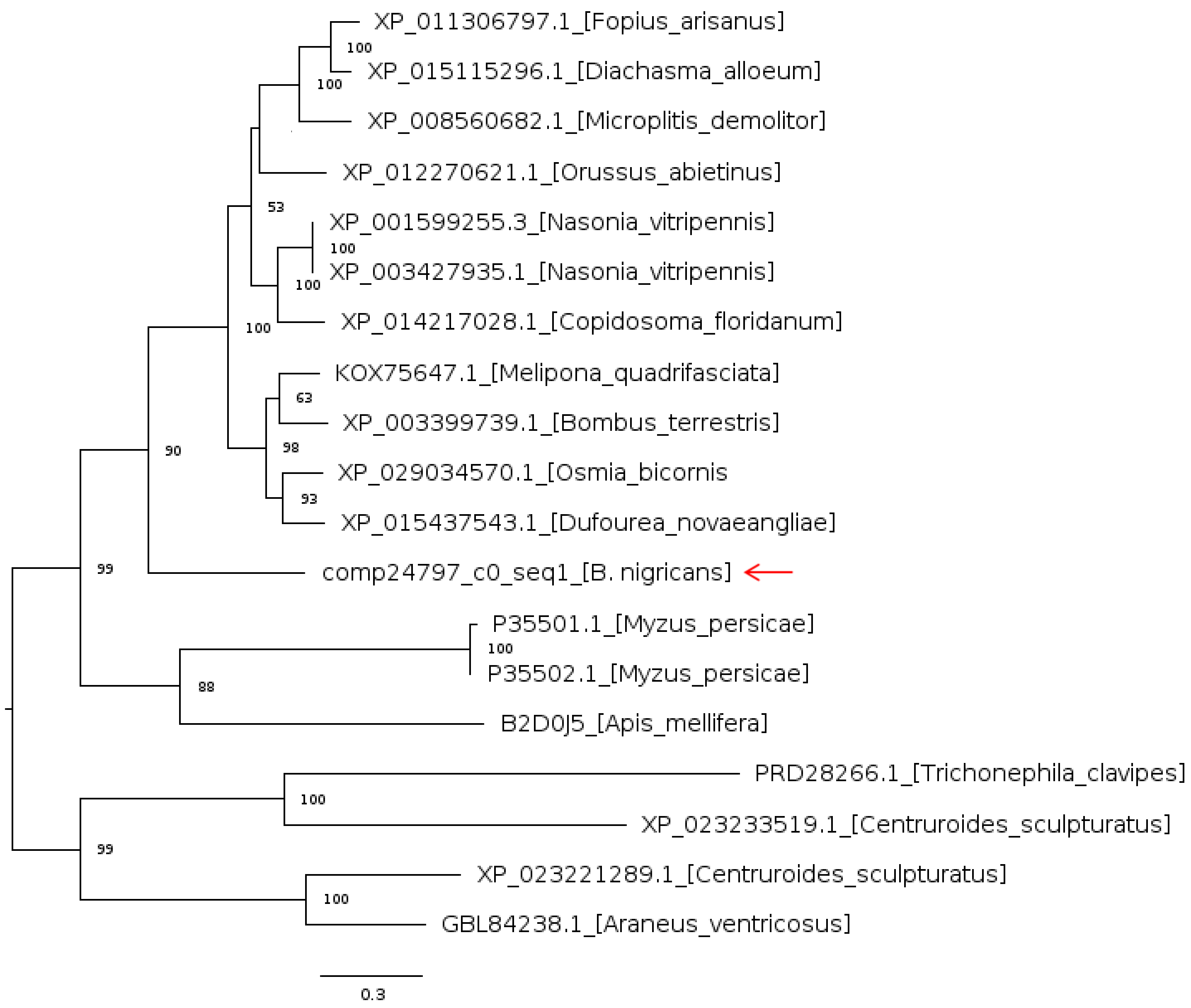

**Fig. S13. Maximum-likelihood tree of serine protease amino acid sequences.** Nodes are labeled with bootstrap support. The accession number of each sequence is followed by the taxon name. Trypsin-like serine protease of *B. nigricans* venom (*Bn*TRY) is indicated by a red arrow. Highlighted branches represent putative homologs of *Bn*TRY in Formicidae (green). Tree is rooted to putative orthologs in Arachnida.

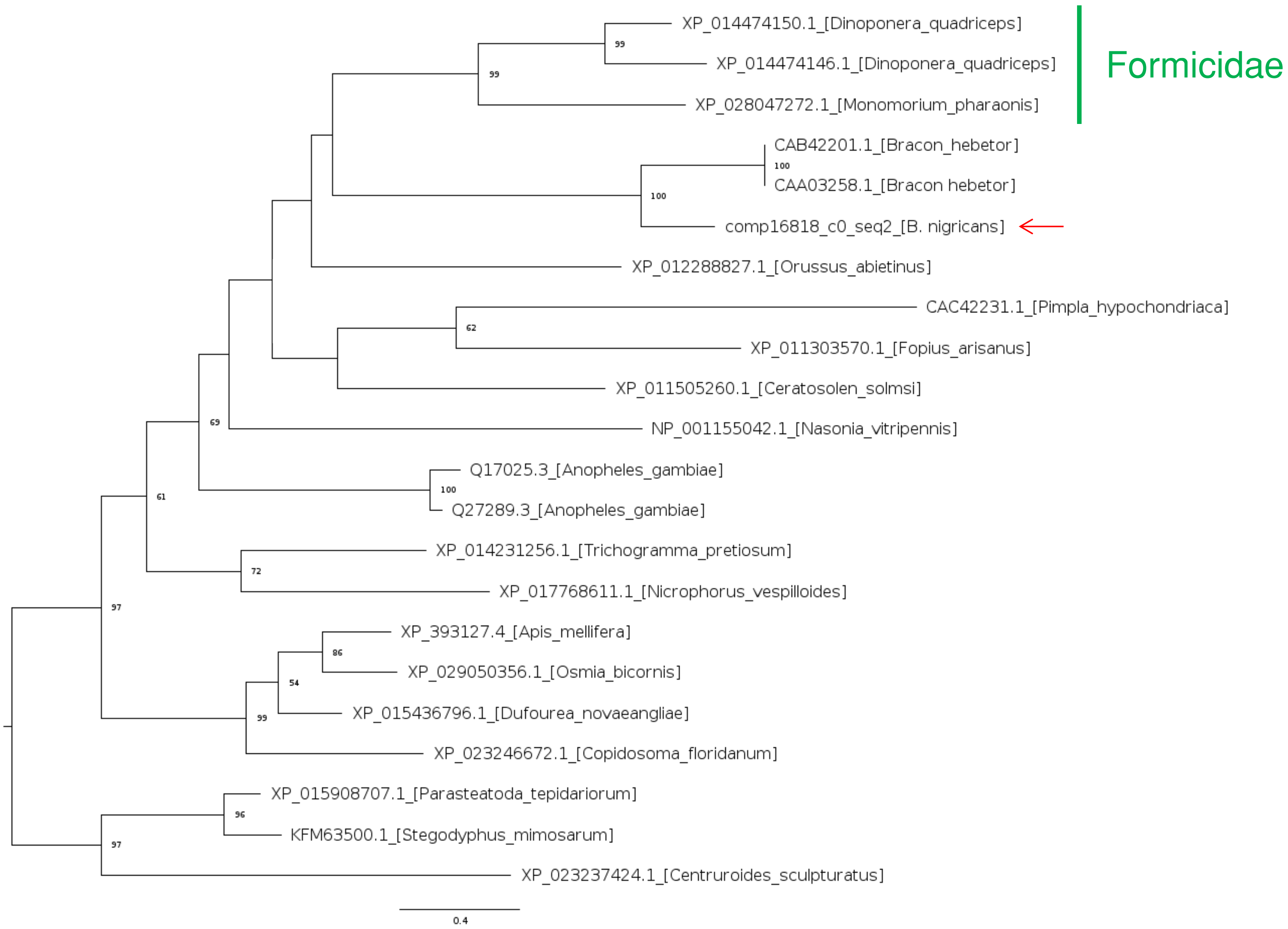

**Fig. S14. Maximum-likelihood tree of aminopeptidase amino acid sequences.** Nodes are labeled with bootstrap support. The accession number of each sequence is followed by the taxon name. Leucyl-cystinyl aminopeptidase of *B. nigricans* venom (*Bn*LCA) is indicated by a red arrow. Highlighted branches represent putative homologs of *Bn*LCA in Braconidae (green). Tree is rooted to putative orthologs in Arachnida.

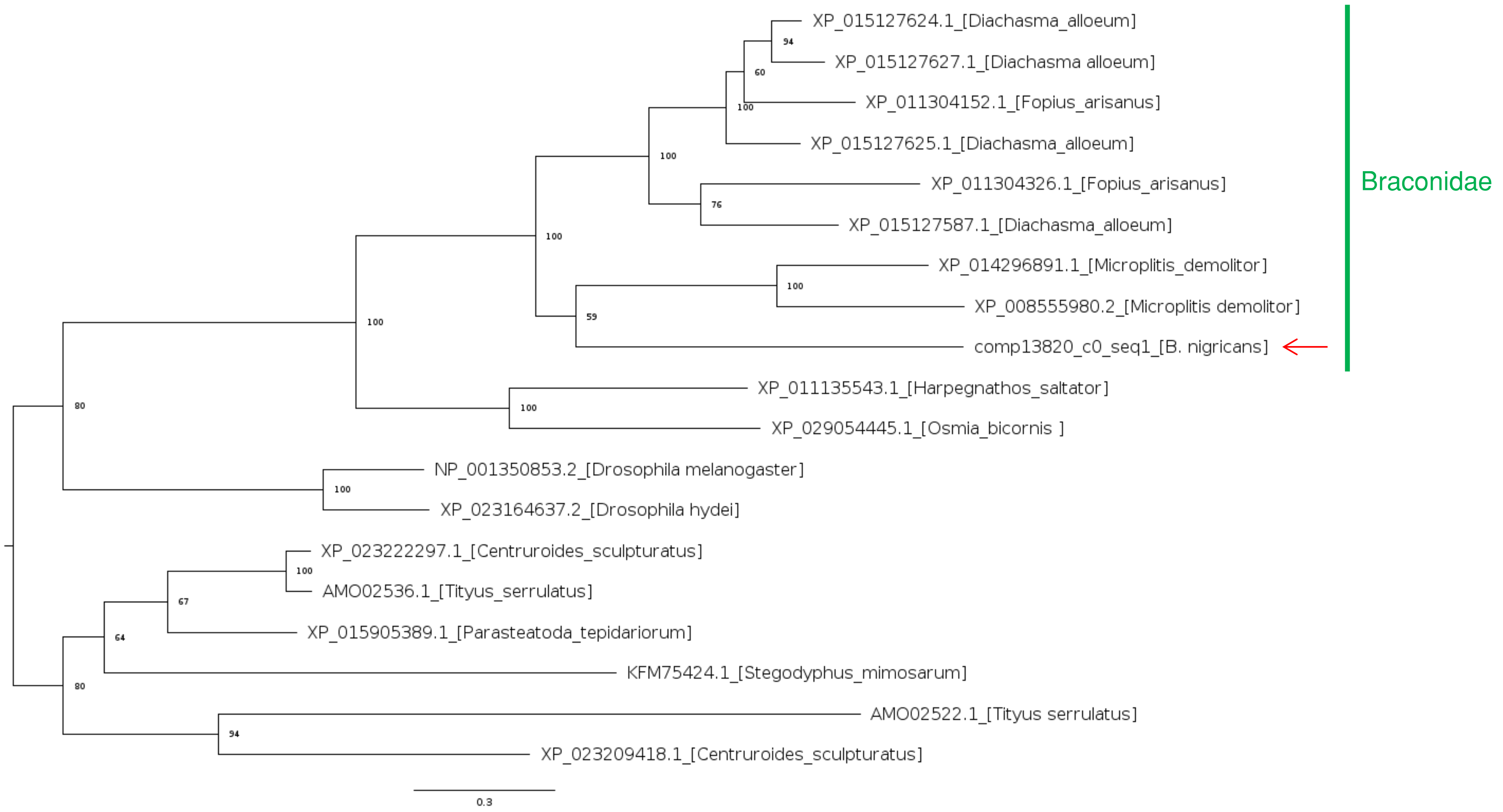

**Fig. S15. Maximum-likelihood tree of mannosidase amino acid sequences.** Nodes are labeled with bootstrap support. The accession number of each sequence is followed by the taxon name. Lysosomal alfa-mannosidase of *B. nigricans* venom (*Bn*LAM) is indicated by a red arrow. Highlighted branches represent putative homologs of *Bn*LAM in Braconidae (green). Tree is rooted to putative orthologs in Arachnida.

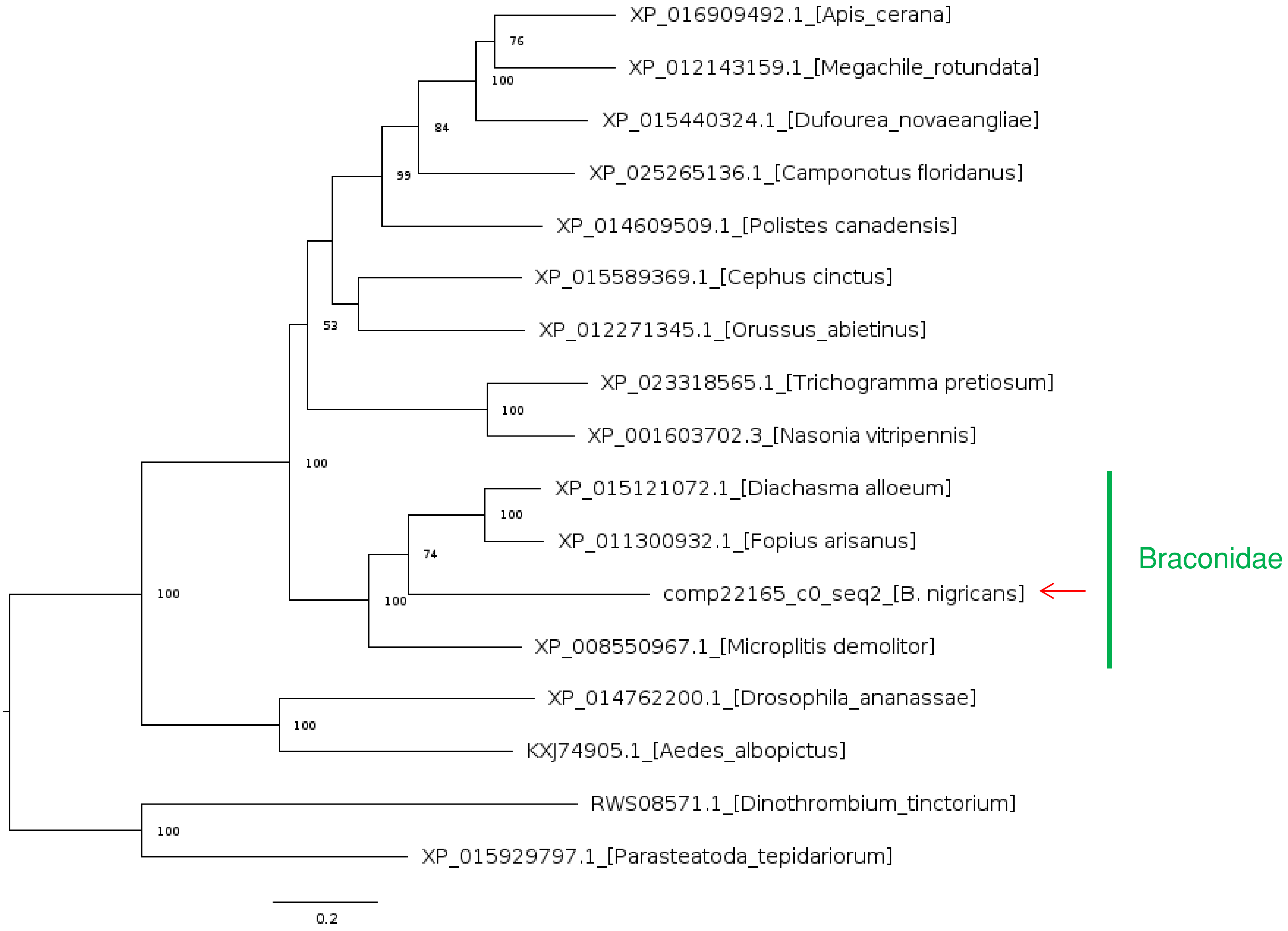

**Fig. S16. Maximum-likelihood tree of odorant-binding protein amino acid sequences.** Nodes are labeled with bootstrap support. The accession number of each sequence is followed by the taxon name. Odorant-binding protein of *B. nigricans* venom (*Bn*OBP) is indicated by a red arrow. Paralogs of *Bn*OBP are indicated by green arrows. Tree is rooted to the putative ortholog in the cat flea *Ctenocephalides felis*.

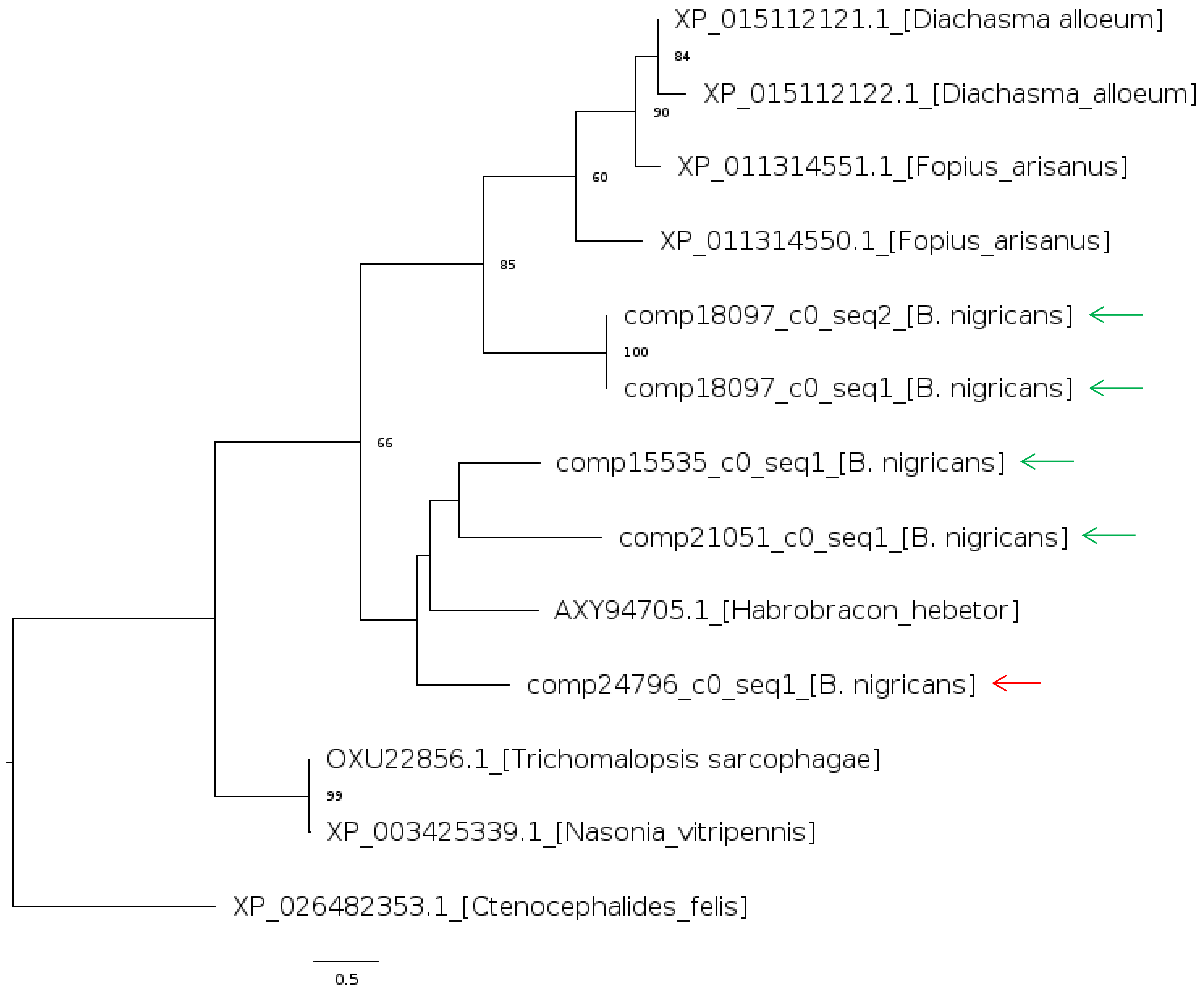

**Fig. S17. Maximum-likelihood tree of protein disulfide-isomerase amino acid sequences.** Nodes are labeled with bootstrap support. The accession number of each sequence is followed by the taxon name. Protein disulfide-isomerase of *B. nigricans* venom (*Bn*PDI) is indicated by a red arrow. Highlighted branches represent putative homologs of *Bn*PDI in Braconidae (green). Tree is rooted to putative orthologs in Arachnida.

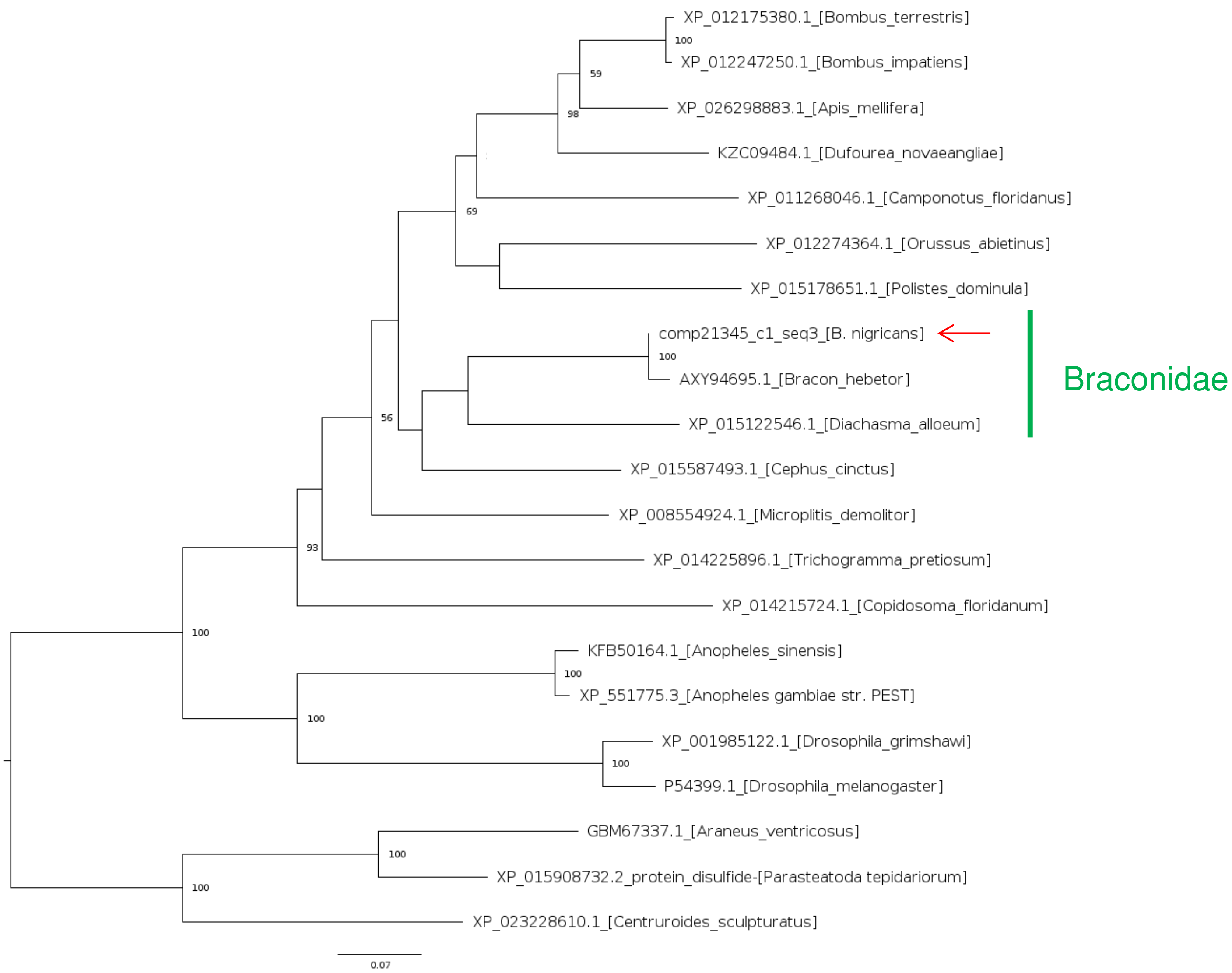

Supplement: Supplementary file 7 — Additional file 7: Figures S10-S17. Phylogenetic trees of most representative B. nigricans venom proteins [file 12864_2019_6396_MOESM7_ESM.pdf]
