# Supplementary material for: Venomics of the ectoparasitoid wasp Bracon nigricans
Source: BMC Genomics. 2020 Jan 10;21:34. doi: 10.1186/s12864-019-6396-4 (PMC6954513; doi:10.1186/s12864-019-6396-4)
Supplement: Supplementary file 8 — Additional file 8: Table S5. Primers used for qRT-PCR analysis of selected venom components [file 12864_2019_6396_MOESM8_ESM.docx]

**Table S5. Primers used for qRT-PCR analysis of selected venom components.**

| Transcript | Sequence |
| --- | --- |
| Phospholipase A2 | F: AAAAGCACGAATCCCTGTATGAAC  R: AGGTTGTGAGCATTGTCCTTCAC |
| Carboxylesterase | F: CCGTGGGCCTTCAACTTCT  R: AGTCCAATGCTCGACCAACAG |
| Trypsin-like serine protease | F: GGGAGGCGTAGTTGGAGTGA  R: AGACGGGCCTCTTCGTGAT |
| Lipase | F: GAAATGCTGCCGCTAATCG  R: AGCTGTAATCTTCTTCAAATCGAACA |
| Leucyl-cystinil aminopeptidase | F: TCAACGTGACAAGAAATTACGAAACT  R: AGCTTATCGGTATCGTCCATCTG |
| Lysosomal alpha-mannosidase | F: AAGCAGATTTATTCTCGGTTGTATTG  R: TGAACACAGATTGTCGAAGCAA |
| Odorant binding protein | F: ATGCCAGGCCAAGTTCGA  R: ATCGCAAATCGTCCTCTGGTT |
| Protein disulphide isomerase | F: TGCTAAGGCTTTCCTGGAGACT  R: AAGCCTCGTCGCTGACAATAC |
| Ribosomal Protein S3  (housekeeping) | F: GACGAATTTCATGCTCTT  R: GTCACTCAGATACAAACTC |
